# Supplementary material for: An efficient class of estimators for finite population mean in the presence of non-response under ranked set sampling (RSS)
Source: PLoS One. 2022 Dec 16;17(12):e0277232. doi: 10.1371/journal.pone.0277232 (PMC9757593; doi:10.1371/journal.pone.0277232)
Supplement: S1 Appendix — (PDF) [file pone.0277232.s001.pdf]

APPENDIX

R-Simulation Commands used.

(1) RSS at second attempt only.

```
set.seed(18)
rho=0.90; sim=20000; k=2; w1=0.60
r=1; m=3 ;n1=9; n=n1+(r*m); rp=round((n-n1)/m,0)
N=1000; w2=1-w1; N1=round(w1*N,0); N2=round((N-N1), 0)
mu1=100; sd1=10; X1=rnorm(N1,mu1,sd1); e1=rnorm(N1,0,1)
Y1=rho*X1+e1*sqrt(1-rho^2); mu2=75; sd2=15
X2=rnorm(N2,mu2,sd2); e2=rnorm(N2,0,1)
Y2=rho*X2+e2*sqrt(1-rho^2); X=c(X1,X2); Y=c(Y1,Y2)
D=data.frame(Y,X); D1=D[1:N1,]; D2=D[(N1+1):(N-(N2/k)),]
DS=rbind(D1,D2); Yb=mean(Y); Xb=mean(X); Vx=var(X)
Vy=var(Y); Vyx=cov(Y,X)
##################################################
yb1=c(); xb1=c(); ybs=c(); xbs=c()
for(i in 1:sim){
s=D1[sample(1:nrow(D1), n1, replace=TRUE),]
yb1=c(yb1, mean(s[,1]))
xb1=c(xb1, mean(s[,2]))
SRS=DS[sample(1:nrow(DS), n, replace=TRUE),]
ybs=c(ybs, mean(s[,1])); xbs=c(xbs, mean(s[,2]))}
##################################################
yb2=c(); xb2=c()
for(k in 1:sim){
rssy=c(); rssx=c()
for(j in 1:rp){
sy=c(); sx=c()
for(i in 1:m){
s=D2[sample(1:nrow(D2), m, replace=TRUE),]
s1=s[order(s[,2]),]; sy=rbind(sy,s1[,1])
sx=rbind(sx,s1[,2])}; rssy=c(rssy,diag(sy))
```

```
rssx=c(rssx,diag(sx))}
yb2=c(yb2, mean(rssy)); xb2=c(xb2, mean(rssx))}
##################################################
yb.r=w1*yb1+w2*yb2; xb.r=w1*xb1+w2*xb2
d1s=Vx*Yb^2/(Yb^2*Vx+Vx*Vy-Vyx^2)
d2s=Vyx*Yb^2/(Yb^2*Vx+Vx*Vy-Vyx^2)
d3s=(1/2)*Vx*(2*Yb-Xb)/(Vx*Yb-Xb*Vyx)
d4s=-(1/2)*(-Yb^2*Xb^3*Vx+2*Yb^2*Xb^3*Vyx+2*Yb^3*Vx^2
-Yb^2*Xb*Vx^2-4*Yb^2*Xb*Vx*Vyx+2*Yb*Xb^2*Vx*Vy+3*Yb*Xb^2*Vx*Vyx
-2*Yb*Xb^2*Vyx^2-Xb^3*Vx*Vy)/(Xb*(Vx*Yb-Xb*Vyx)^2)
d5s=(1/8)*Yb^2*Vx*(8*Xb^2-Vx)/(Xb^2*(Yb^2*Vx+Vx*Vy-Vyx^2))
d6s=-(1/8)*Yb*(4*Yb^2*Xb^2*Vx-8*Yb*Xb^3*Vyx-Yb^2*Vx^2+
Yb*Xb*Vyx*Vx-4*Xb^2*Vy*Vx+4*Xb^2*Vyx^2)/(Xb^3*(Yb^2*Vx
+Vx*Vy-Vyx^2))
# Diana SRS estimators
trao.s=d1s*ybs+d2s*(Xb-xbs)
tratio.s=(d1s*ybs+d2s*(Xb-xbs))*(Xb/xbs)
texp.s=(d1s*ybs+d2s*(Xb-xbs))*exp((Xb-xbs)/(Xb+xbs))
mrao.s=(1/sim)*sum((trao.s-Yb)^2)
mratio.s=(1/sim)*sum((tratio.s-Yb)^2)
mexp.s=(1/sim)*sum((texp.s-Yb)^2)
MSE.s=c(mrao.s, mratio.s, mexp.s)
##############################################
#Under RSS
d1rss=Vx*Yb^2/(Yb^2*Vx+Vx*Vy-Vyx^2)
d2rss=Vyx*Yb^2/(Yb^2*Vx+Vx*Vy-Vyx^2)
d3rss=(1/2)*Vx*(2*Yb-Xb)/(Vx*Yb-Xb*Vyx)
d4rss=-(1/2)*(-Yb^2*Xb^3*Vx+2*Yb^2*Xb^3*Vyx+2*Yb^3*Vx^2
-Yb^2*Xb*Vx^2-4*Yb^2*Xb*Vx*Vyx+2*Yb*Xb^2*Vx*Vy
+3*Yb*Xb^2*Vx*Vyx
-2*Yb*Xb^2*Vyx^2-Xb^3*Vx*Vy)/(Xb*(Vx*Yb-Xb*Vyx)^2)
d5rss=(1/8)*Yb^2*Vx*(8*Xb^2-Vx)/(Xb^2*(Yb^2*Vx+Vx*Vy-Vyx^2))
d6rss=-(1/8)*Yb*(4*Yb^2*Xb^2*Vx-8*Yb*Xb^3*Vyx-Yb^2*Vx^2
+Yb*Xb*Vyx*Vx-4*Xb^2*Vy*Vx+4*Xb^2*Vyx^2)/(Xb^3*(Yb^2*Vx
+Vx*Vy-Vyx^2))
# Proposed Estimators
trao.r=d1rss*yb.r+d2rss*(Xb-xb.r)
tratio.r=(d1rss*yb.r+d2rss*(Xb-xb.r))*(Xb/xb.r)
texp.r=(d1rss*yb.r+d2rss*(Xb-xb.r))*exp((Xb-xb.r)/(Xb+xb.r))
mrao.r=(1/sim)*sum((trao.r-Yb)^2)
mratio.r=(1/sim)*sum((tratio.r-Yb)^2)
mexp.r=(1/sim)*sum((texp.r-Yb)^2)
MSE.r=c(mrao.r, mratio.r, mexp.r); Est=c("Rao","Ratio","Exp")
RE=round(MSE.s/MSE.r,3)
Results=cbind(Est, MSE.s, MSE.r, RE); Results
```

(1) RSS at both attempt.

```
set.seed(18)
rho=0.90; sim=20000; k=2; w1=0.60
r=1; m=3 ; n1=9; n=n1+(r*m); rp=n/m
N=1000; w2=1-w1; N1=round(w1*N,0); N2=round((N-N1), 0)
mu1=100; sd1=10; X1=rnorm(N1,mu1,sd1); e1=rnorm(N1,0,1)
Y1=rho*X1+e1*sqrt(1-rho^2); mu2=75; sd2=15
```

```
X2=rnorm(N2,mu2,sd2); e2=rnorm(N2,0,1)
Y2=rho*X2+e2*sqrt(1-rho^2);X=c(X1,X2); Y=c(Y1,Y2)
D=data.frame(Y,X); D1=D[1:N1,]; D2=D[(N1+1):(N-(N2/k)),]
DS=rbind(D1,D2); Yb=mean(Y); Xb=mean(X); Vx=var(X)
Vy=var(Y); Vyx=cov(Y,X)
##################################################
ybs=c(); xbs=c()
for(i in 1:sim){
SRS=DS[sample(1:nrow(DS), n, replace=TRUE),]
ybs=c(ybs, mean(SRS[,1])); xbs=c(xbs, mean(SRS[,2]))}
###################################################
yb1=c(); xb1=c(); yb2=c(); xb2=c()
for(k in 1:sim){
rssy1=c(); rssx1=c(); rssy2=c(); rssx2=c()
for(j in 1:rp){
sy1=c(); sx1=c(); sy2=c(); sx2=c()
for(i in 1:m){
s=D1[sample(1:nrow(D1), m, replace=TRUE),]
s1=s[order(s[,2]),]; sy1=rbind(sy1,s1[,1])
sx1=rbind(sx1,s1[,2])
s2=D2[sample(1:nrow(D2), m, replace=TRUE),]
s3=s2[order(s2[,2]),]; sy2=rbind(sy2,s3[,1])
sx2=rbind(sx2,s3[,2])}
rssy1=c(rssy1,diag(sy1)); rssx1=c(rssx1,diag(sx1))
rssy2=c(rssy2,diag(sy2)); rssx2=c(rssx2,diag(sx2))}
yb1=c(yb1, mean(rssy1)); xb1=c(xb1, mean(rssx1))
yb2=c(yb2, mean(rssy2)); xb2=c(xb2, mean(rssx2))}
##################################################
yb.r=w1*yb1+w2*yb2; xb.r=w1*xb1+w2*xb2
d1srs=(Yb^2*Vx)/(Yb^2*Vx+Vx*Vy-Vyx^2)
d2srs=(Yb^2*Vyx)/(Yb^2*Vx+Vx*Vy-Vyx^2)
d3srs=(1/2)*Vx*(2*Yb-Xb)/(Vx*Yb-Xb*Vyx)
d4srs=-(1/2)*(-Yb^2*Xb^3*Vx+2*Yb^2*Xb^3*Vyx+2*Yb^3*Vx^2
-Yb^2*Xb*Vx^2-4*Yb^2*Xb*Vx*Vyx+2*Yb*Xb^2*Vx*Vy+3*Yb*Xb^2
*Vx*Vyx-2*Yb*Xb^2*Vyx^2-Xb^3*Vx*Vy)/(Xb*(Vx*Yb-Xb*Vyx)^2)
d5srs=(1/8)*Yb^2*Vx*(8*Xb^2-Vx)/(Xb^2*(Yb^2*Vx+Vx*Vy-Vyx^2))
d6srs=-(1/8)*Yb*(4*Yb^2*Xb^2*Vx-8*Yb*Xb^3*Vyx-Yb^2*Vx^2+Yb*Xb
*Vyx*Vx-4*Xb^2*Vy*Vx+4*Xb^2*Vyx^2)/(Xb^3*(Yb^2*Vx+Vx*Vy-Vyx^2))
# Diana SRS estimators
trao.s=d1srs*ybs+d2srs*(Xb-xbs)
tratio.s=(d1srs*ybs+d2srs*(Xb-xbs))*(Xb/xbs)
texp.s=(d1srs*ybs+d2srs*(Xb-xbs))*exp((Xb-xbs)/(Xb+xbs))
mrao.s=(1/sim)*sum((trao.s-Yb)^2)
mratio.s=(1/sim)*sum((tratio.s-Yb)^2)
mexp.s=(1/sim)*sum((texp.s-Yb)^2)
MSE.s=c(mrao.s, mratio.s, mexp.s)
##################################################
d1rss=(Yb^2*Vx)/(Yb^2*Vx+Vx*Vy-Vyx^2)
d2rss=(Yb^2*Vyx)/(Yb^2*Vx+Vx*Vy-Vyx^2)
d3rss=(1/2)*Vx*(2*Yb-Xb)/(Vx*Yb-Xb*Vyx)
d4rss=-(1/2)*(-Yb^2*Xb^3*Vx+2*Yb^2*Xb^3*Vyx+2*Yb^3*Vx^2-Yb^2*
Xb*Vx^2-4*Yb^2*Xb*Vx*Vyx+2*Yb*Xb^2*Vx*Vy+3*Yb*Xb^2*Vx*Vyx-2
```

```
*Yb*Xb^2*Vyx^2-Xb^3*Vx*Vy)/(Xb*(Vx*Yb-Xb*Vyx)^2)
d5rss=(1/8)*Yb^2*Vx*(8*Xb^2-Vx)/(Xb^2*(Yb^2*Vx+Vx*Vy-Vyx^2))
d6rss=-(1/8)*Yb*(4*Yb^2*Xb^2*Vx-8*Yb*Xb^3*Vyx-Yb^2*Vx^2+Yb*Xb
*Vyx*Vx-4*Xb^2*Vy*Vx+4*Xb^2*Vyx^2)/(Xb^3*(Yb^2*Vx+Vx*Vy-Vyx^2))
# Proposed
trao.r=d1rss*yb.r+d2rss*(Xb-xb.r)
tratio.r=(d1rss*yb.r+d2rss*(Xb-xb.r))*(Xb/xb.r)
texp.r=(d1rss*yb.r+d2rss*(Xb-xb.r))*exp((Xb-xb.r)/(Xb+xb.r))
mrao.r=(1/sim)*sum((trao.r-Yb)^2)
mratio.r=(1/sim)*sum((tratio.r-Yb)^2)
mexp.r=(1/sim)*sum((texp.r-Yb)^2)
MSE.r=c(mrao.r, mratio.r, mexp.r); Est=c("Rao","Ratio","Exp")
RE=round(MSE.s/MSE.r,3); Results=cbind(Est, MSE.s, MSE.r, RE)
Results
```
