# Supplementary material for: An efficient class of estimators for finite population mean in the presence of non-response under ranked set sampling (RSS)
Source: PLoS One. 2022 Dec 16;17(12):e0277232. doi: 10.1371/journal.pone.0277232 (PMC9757593; doi:10.1371/journal.pone.0277232)
Supplement: Attachment — Submitted filename: PONE-D-22-15506_R1_Comments.pdf [file pone.0277232.s003.pdf]

PLOS ONE

An efficient class of estimators for finite population mean in the presence of non-response under ranked set sampling (RSS)

--Manuscript Draft--

**Manuscript Number:**	PONE-D-22-15506R1
**Article Type:**	Research Article
**Full Title:**	An efficient class of estimators for finite population mean in the presence of non-response under ranked set sampling (RSS)
**Short Title:**	Efficient class of estimators in the presence of non-response under RSS
**Corresponding Author:**	Syed Abdul Rehman, PhD<br>Balochistan University of Information Technology Engineering and Management Sciences<br>Quetta, Pakistan PAKISTAN
**Keywords:**	Ranked set sampling, auxiliary variable, non-response, Monte Carlo simulation
**Abstract:**	In this study, we address the problem of estimating the finite population mean when the non-response occurs on the characteristics under study. We propose a class of Rao-regression type estimators when ranked set sampling (RSS) procedure is used to collect the data from non-response group only and from both, the response and non-response groups. The information provided by auxiliary variable is used at both stages i.e., at data collection stage and at the estimation stage. Expressions for bias and mean square error of the estimators are obtained up to first order of approximation and comprehensive simulation study is carried out to observe the performances of the proposed estimators under non-response.
**Order of Authors:**	Syed Abdul Rehman, PhD
	Javid Shabbir, PhD
**Response to Reviewers:**	The link of "View Decision Letter" is blank and blocked, however Response to the reviewer file is uploaded.
**Additional Information:**	
**Question**	**Response**
**Financial Disclosure**<br><br>Enter a financial disclosure statement that describes the sources of funding for the work included in this submission. Review the submission guidelines for detailed requirements. View published research articles from *PLOS ONE* for specific examples.<br><br>This statement is required for submission and **will appear in the published article** if the submission is accepted. Please make sure it is accurate.	The authors received no specific funding for this work

Powered by Editorial Manager® and ProduXion Manager® from Aries Systems Corporation

**Unfunded studies**<br>Enter: *The author(s) received no specific funding for this work.*<br><br>**Funded studies**<br>Enter a statement with the following details:<br>• Initials of the authors who received each award<br>• Grant numbers awarded to each author<br>• The full name of each funder<br>• URL of each funder website<br>• Did the sponsors or funders play any role in the study design, data collection and analysis, decision to publish, or preparation of the manuscript?<br>• **NO** - Include this sentence at the end of your statement: *The funders had no role in study design, data collection and analysis, decision to publish, or preparation of the manuscript.*<br>• **YES** - Specify the role(s) played.<br><br>* typeset	
**Competing Interests**<br><br>Use the instructions below to enter a competing interest statement for this submission. On behalf of all authors, disclose any competing interests that could be perceived to bias this work—acknowledging all financial support and any other relevant financial or non-financial competing interests.<br><br>This statement is required for submission and **will appear in the published article** if the submission is accepted. Please make sure it is accurate and that any funding sources listed in your Funding Information later in the submission form are also declared in your Financial Disclosure statement.<br><br>View published research articles from *PLOS ONE* for specific examples.	The authors have declared that no competing interests exist.

**NO authors have competing interests**<br><br>Enter: *The authors have declared that no competing interests exist.*<br><br>**Authors with competing interests**<br><br>Enter competing interest details beginning with this statement:<br><br>*I have read the journal's policy and the authors of this manuscript have the following competing interests: [insert competing interests here]*<br><br>* typeset	
**Ethics Statement**<br><br>Enter an ethics statement for this submission. This statement is required if the study involved:<br><br>• Human participants<br>• Human specimens or tissue<br>• Vertebrate animals or cephalopods<br>• Vertebrate embryos or tissues<br>• Field research<br><br>Write "N/A" if the submission does not require an ethics statement.<br><br>General guidance is provided below. Consult the submission guidelines for detailed instructions. **Make sure that all information entered here is included in the Methods section of the manuscript.**	This material is the authors' own original work, which has not been previously published elsewhere. 2) The paper is not currently being considered for publication elsewhere. 3) The paper reflects the authors' own research and analysis in a truthful and complete manner.

Powered by Editorial Manager® and ProduXion Manager® from Aries Systems Corporation

**Format for specific study types**<br><br>**Human Subject Research (involving human participants and/or tissue)**<br>• Give the name of the institutional review board or ethics committee that approved the study<br>• Include the approval number and/or a statement indicating approval of this research<br>• Indicate the form of consent obtained (written/oral) or the reason that consent was not obtained (e.g. the data were analyzed anonymously)<br><br>**Animal Research (involving vertebrate animals, embryos or tissues)**<br>• Provide the name of the Institutional Animal Care and Use Committee (IACUC) or other relevant ethics board that reviewed the study protocol, and indicate whether they approved this research or granted a formal waiver of ethical approval<br>• Include an approval number if one was obtained<br>• If the study involved *non-human primates*, add *additional details* about animal welfare and steps taken to ameliorate suffering<br>• If anesthesia, euthanasia, or any kind of animal sacrifice is part of the study, include briefly which substances and/or methods were applied<br><br>**Field Research**<br><br>Include the following details if this study involves the collection of plant, animal, or other materials from a natural setting:<br>• Field permit number<br>• Name of the institution or relevant body that granted permission	
**Data Availability**<br><br>Authors are required to make all data underlying the findings described fully available, without restriction, and from the time of publication. PLOS allows rare exceptions to address legal and ethical concerns. See the PLOS Data Policy and FAQ for detailed information.	Yes - all data are fully available without restriction

A Data Availability Statement describing where the data can be found is required at submission. Your answers to this question constitute the Data Availability Statement and **will be published in the article**, if accepted.<br><br>**Important:** Stating ‘data available on request from the author’ is not sufficient. If your data are only available upon request, select ‘No’ for the first question and explain your exceptional situation in the text box.<br><br>Do the authors confirm that all data underlying the findings described in their manuscript are fully available without restriction?	
**Describe where the data may be found in full sentences. If you are copying our sample text, replace any instances of XXX with the appropriate details.**<br><br>• If the data are **held or will be held in a public repository**, include URLs, accession numbers or DOIs. If this information will only be available after acceptance, indicate this by ticking the box below. For example: *All XXX files are available from the XXX database (accession number(s) XXX, XXX.*).<br>• If the data are all contained **within the manuscript and/or Supporting Information files**, enter the following: *All relevant data are within the manuscript and its Supporting Information files.*<br>• If neither of these applies but you are able to provide **details of access elsewhere**, with or without limitations, please do so. For example:<br><br>*Data cannot be shared publicly because of [XXX]. Data are available from the XXX Institutional Data Access / Ethics Committee (contact via XXX) for researchers who meet the criteria for access to confidential data.*<br><br>*The data underlying the results presented in the study are available from (include the name of the third party*	The data analysed in this study is generated by authors on R softwares which has no copy rights with any other author.

and contact information or URL).<br>• This text is appropriate if the data are owned by a third party and authors do not have permission to share the data.<br><br>* typeset	
Additional data availability information:	Tick here if the URLs/accession numbers/DOIs will be available only after acceptance of the manuscript for publication so that we can ensure their inclusion before publication.

Powered by Editorial Manager® and ProduXion Manager® from Aries Systems Corporation

Manuscript
Click here to access/download;Manuscript;SyedAbdulRehman.pdf

An efficient class of estimators for finite population mean in the presence of non-response under ranked set sampling (RSS)

Syed Abdul Rehman[1]◑, Javid Shabbir[2,3]◑

1 Department of Mathematical Sciences, Balochistan University of Information Technology, Engineering and Management Sciences, Quetta, Pakistan
2 Department of Statistics, Quaid-i-Azam university, Islamabad, Pakistan
3 Department of Statistics, University of Wah, Wah cantt, Islamabad, Pakistan

◑These authors contributed equally to this work.
‡These authors also contributed equally to this work.
* rehmanbukhari725@gmail.com

Abstract

In this study, we address the problem of estimating the finite population mean when the non-response occurs on the characteristics under study. We propose a class of Rao-regression type estimators when ranked set sampling (RSS) procedure is used to collect the data from non-response group only and from both, the response and non-response groups. The information provided by auxiliary variable is used at both stages i.e., at data collection stage and at the estimation stage. Expressions for bias and mean square error of the estimators are obtained up to first order of approximation and comprehensive simulation study is carried out to observe the performances of the proposed estimators under non-response.
Keywords: Ranked set sampling, auxiliary variable, non-response, Monte Carlo simulation

Author summary

Syed Abdul Rehman is PhD scholar in statistics and he started publishing his research in 2021 and published several papers in the same year. his research area is non-response in surveys, ranked set sampling and simulation studies. One of his paper has recieved maximum views and downloads on the concerned journal website.

Javid Shabbir is professor of Statistics at university of Wah currently. His research area is aa wide range of topics in the field of survey sampling and estimation. He has supervised 20 Phd students during his services at Quaid-iAzam university, Islamabad, Pakistan. he is also editor of a well recognized journal in mathematics and statistics.

Introduction

A survey can be conducted using a variety of sampling techniques depending upon the situation and structure of data. Simple random sampling (SRS) is an easy and unbiased data collection method, but in situations where the overall population is scattered and diverse, it requires a larger sample for a given margin of accuracy. On the other hand, estimation of population parameters becomes more problematic if non-response occurs in the data collected from such a scattered population. Non-response is an inability to

collect data from one or more units of population units selected in a sample frame. Most of the time, we have to generalize the survey results by using a smaller sample which demands that an appropriate sampling method should be employed to conduct a sample which represent the population well enough. In such cases, ranked set sampling (RSS) is an alternative and more efficient technique to collect a sample since it is determined by ranking a greater number of sampling units based on their relative sizes, then picking a smaller number of units from each ranked group under observation. As a result, RSS increases the precision of estimates by reducing the sampling errors. The availability of some appropriate auxiliary variables that are correlated with the variable under study is also a factor in improving the estimation. RSS sampling procedure utilizes the auxiliary variable information to collect a data by using its order at the data collection stage.

Theory of RSS was first introduced by McIntyre (1952). In this method, units of the study variable are ordered by either visual judgment or by some cheap quantitative measures. Takahasi & Wakimoto (1968) developed an unbiased estimator of the population mean under RSS technique. Lynne Stokes (1977) exposed another important use of the auxiliary variable, and it is that we can use the ranks of to order the units of . Singh et al. (2014), Abu-Dayyeh et al. (2003), Kadilar & Cingi (2005), Malik & Singh (2013), Singh & Sharma (2015), Fatima et al. (2022) and Muneer et al. (2017) and Rehman & Shabbir (2021) can also be seen for the use of this feature of auxiliary variable. The use of an auxiliary variable can also be used in reducing sample variation and increasing efficiency of estimates at the estimation stage. Several authors have claimed that the use of auxiliary variables at data collection stage can result into more efficient estimates. It's worth noting that adding more auxiliary variables at estimation stage improves the efficiency of estimates.

Bouza-Herrera (2013) has considered the comprehensive layout of the non-response under RSS. In his book, the problem of non-response when the sample is collected through RSS on the second attempt from a non-response group can be seen. Inspired by Bouza-Herrera (2013) and Al-Omari & Bouza (2015), Diana et al. (2011) and Riaz et al. (2014), we investigate the estimation of finite population mean under RSS in the presence of non-response.

Importance of the problem

The non-response is thought as one of the most important problems in theory of survey and data analysis. The occurrence of non-response in a data lead to skewed estimates and less representative sample of the population. The discrepancy between respondents and non-respondents on a given measure combined with the non-response rate in the population produces non-response bias. A lower response rate raises the risk of larger non-response bias, but when data is missing at random (MAR), a lower response rate has no effect on the non-response error. In practice, using information from an auxiliary variable is often costly, but classifying things based on it is quite straightforward. We assume that RSS sampling technique may improve the precision in estimating population mean by using the information of auxiliary variable at estimation stage.

Methodology

We consider the naive model of Hansen & Hurwitz (1946). According to which, we draw a sample S of size n_s by using SRSWOR method from a finite population Ω of size N. Let n_1 units respond to the survey at first attempt while $n_2\,(= n - n_1)$ units do not respond. Special efforts are made to approach the non-responding units and a part of them $(n_2' = n_2/k; k > 1)$ is included in the sample. Thus, we get a final sample of size $n = n_1 + n_2'$ for estimation purpose. This allows the entire population is to be separated

into two complementary groups called response and non-response group.

Let $(Y_{ji}, X_{ji})_{i=1}^{N_j}$; $j = 1, 2$ shows population units of study variable (Y) and auxiliary variable (X) in the two groups. our goal is to estimate the finite population mean $\left(\bar{Y}\right)$ of the study variable. Hansen & Hurwitz (1946) suggested the following unbiased estimator when non-response occurs on Y.

$$\bar{y}^*_{srs} = w_1 \bar{y}_1 + w_2 \bar{y}'_2 \tag{1}$$

The variance of $\bar{y}^*_{srs}$ is given by,

$$Var\left(\bar{y}^*_{srs}\right) = \left(\frac{1-\lambda}{n}\right)\sigma_y^2 + \frac{W_2\left(K-1\right)}{n}\sigma_{y2}^2 = V_{ys} \tag{2}$$

where

$\bar{y}_1 = \frac{1}{n_1}\sum\limits_{i=1}^{n_1} y_{1i}, \quad \bar{y}'_2 = \frac{1}{n'_2}\sum\limits_{i=1}^{n'_2} y'_{2i}, \quad \lambda = \frac{n}{N}, \quad w_j = \frac{n_j}{n}; \quad j = 1, 2$

and

$\bar{Y}_1 = \frac{1}{N_1}\sum\limits_{i=1}^{N_1} Y_{1i}, \quad \bar{Y}_2 = \frac{1}{N_2}\sum\limits_{i=1}^{N_2} Y_{2i}, \quad \bar{Y} = \frac{1}{N}\sum\limits_{i=1}^{N} Y_i = W_1\bar{Y}_1 + W_2\bar{Y}_2, \quad W_j = \frac{N_j}{N}$

$\sigma_y^2 = \frac{1}{N-1}\sum\limits_{i=1}^{N}\left(Y_i - \bar{Y}\right)^2, \quad \sigma_{y2}^2 = \frac{1}{N_2-1}\sum\limits_{i=1}^{N}\left(Y_{2i} - \bar{Y}_2\right)^2$

Similar results can be obtained for an auxiliary variable X when it is involved in the estimation of population mean for the study variable Y with the following covariance,

$$Cov\left(\bar{y}^*_{srs}, \bar{x}^*_{srs}\right) = \frac{1-\lambda}{n}S_{yx} - \frac{W_2\left(K-1\right)}{n}S_{yx2}^2 = V_{yxs}$$

When the population mean of the auxiliary variable is known and incomplete information exist only on the study variable, then the Rao (1986) and Rao (1987) suggested the following ratio and regression estimators for estimating $\bar{Y}$ as,

$$\bar{y}_{ratio} = \bar{y}^*\left(\frac{\bar{X}}{\bar{x}}\right) \tag{3}$$

and

$$\bar{y}_{reg} = \bar{y}^* + \hat{\beta}_{yx}\left(\bar{X} - \bar{x}\right) \tag{4}$$

Cochran (1977) and Khare & Srivastava (1997) defined the following ratio and regression estimators when non-response occurs on both variables,

$$\bar{y}^*_{ratio} = \bar{y}^*\left(\frac{\bar{X}}{\bar{x}^*}\right) \tag{5}$$

and

$$\bar{y}^*_{reg} = \bar{y}^* + \hat{\beta}^*_{yx}\left(\bar{X} - \bar{x}^*\right) \tag{6}$$

where $\hat{\beta}_{yx} = \frac{s_{yx}}{s_x^2}$ and $\hat{\beta}^*_{yx} = \frac{s^*_{yx}}{s_x^{*2}}$ are estimates of population regression coefficient $\beta_{yx} = \frac{S_{yx}}{S_x^2}$ of Y on X such that

$$s_{yx} = \frac{\left(\sum\limits_{i=1}^{n_1} y_{1i}x_{1i} + k\sum\limits_{i=1}^{n'_2} y'_{2i}x'_{2i} - n\bar{y}^*\bar{x}\right)}{n-1}, \quad s_x^2 = \frac{\left(\sum\limits_{i=1}^{n_1} x_{1i}^2 + k\sum\limits_{i=1}^{n'_2} x_{2i}^{'2} - n\bar{x}^2\right)}{n-1}$$

$$s^*_{yx} = \frac{\left(\sum\limits_{i=1}^{n_1} y_{1i}x_{1i} + k\sum\limits_{i=1}^{n'_2} y'_{2i}x'_{2i} - n\bar{y}^*\bar{x}^*\right)}{n-1}, \quad s_x^{*2} = \frac{\left(\sum\limits_{i=1}^{n_1} x_{1i}^2 + k\sum\limits_{i=1}^{n'_2} x_{2i}^{'2} - n\bar{x}^{*2}\right)}{n-1}$$

where

$$\bar{x}^* = w_1\bar{x}_1 + w_2\bar{x}_2' \tag{7}$$

Riaz et al. (2014) proposed the following Rao-regression type estimators in the way of Rao (1991).

$$t_{s1} = d_1\bar{y}^* + d_2\left(\bar{X} - \bar{x}^*\right) \tag{8}$$

where d_1 and d_2 are scalars, whose values are either pre-determined or calculated wisely to minimize the MSE of the estimator. The minimum MSE of t_{s1} with the optimum values of d_1 and d_2 is given by,

$$d_{1opt} = \frac{\bar{Y}^2 V_{xs}}{\bar{Y}^2 V_{xs} + V_{xs}V_{ys} - \rho_{yx}V_{yx}V_{xs}}, \quad d_{2opt} = \frac{\bar{Y}^2 \rho_{yx}\sqrt{V_{yx}V_{xs}}}{\bar{Y}^2 V_{xs} + V_{xs}V_{ys} - \rho_{yx}V_{yx}V_{xs}}$$

and

$$MSE(t_{s1})_{\min} = \frac{\bar{Y}^2\left(V_{xs}V_{ys} - \rho_{yx}V_{yx}\right)}{\bar{Y}^2 V_{xs} + V_{xs}V_{ys} - \rho_{yx}V_{yx}} \tag{9}$$

Riaz et al. (2014) also proposed the following generalized class of Rao-regression type estimators by using the idea of Diana et al. (2011),

$$t_{si} = \left(d_3\bar{y}^* + d_4\bar{u}\right)h\left(\bar{u}\right), \tag{10}$$

where d_3 and d_4 are constants and h is generic function of $\bar{u} = \bar{X} - \bar{x}^*$ satisfying some mild conditions. The optimum values of d_3 and d_4 along with the minimum MSE are given by,

$$d_{3opt} = \frac{\bar{Y}^2 V_{xs}\left(a^2 - 2b^2 V_{xs} + acV_{xs}\right)}{a\left[a^2\left(\bar{Y}^2 V_{xs} + V_{xs}V_{ys} - V_{yxs}^2\right) + \bar{Y}^2\left(2ac - 3b^2\right)V_{xs}^2\right]}$$

$$d_{4opt} = \frac{\bar{Y}\left[a^3\bar{Y}V_{yxs} + G - 2ab^2\bar{Y}V_{xs}V_{yxs} + b^3\bar{Y}^2V_{xs}^2\right]}{a^2\left[a^2\left(\bar{Y}^2 V_{xs} + V_{xs}V_{ys} - V_{yxs}^2\right) + \bar{Y}^2\left(2ac - 3b^2\right)V_{xs}^2\right]}$$

$$MSE(t_{si})_{\min} = \frac{\bar{Y}^2\left[a^4V_{ys}^2\left(V_{ys}V_{xs} - V_{yxs}^2\right) - H + 2ab^2c\bar{Y}^2V_{xs}^4 - b^4\bar{Y}^2V_{xs}^4\right]}{a^2\left[a^2\left(\bar{Y}^2 V_{xs} + V_{xs}V_{ys} - V_{yxs}^2\right) + \bar{Y}^2V_{xs}^2\left(2ac - 3b^2\right)\right]} \tag{11}$$

where

$G = a^2\left\{c\bar{Y}V_{xs}V_{yxs} - b\left(\bar{Y}^2V_{xs} - V_{ys}V_{xs} + V_{yxs}^2\right)\right\}$

$H = a^2V_{xs}\left\{b^2\left(V_{ys}V_{xs} - V_{yxs}^2\right) + c^2\bar{Y}^2V_{xs}^2\right\}$

Riaz et al. (2014) showed that this class of estimators is more precise than usual Hansen & Hurwitz (1946), ratio and regression estimator as discussed above under when some certain conditions are satisfied. It should be noted that the values of a, b and c are $\{1, -1, 1\}$ and $\left\{1, -\frac{1}{2}, \frac{3}{8}\right\}$ for $h\left(\bar{u}\right) = \left(\frac{\bar{X}}{\bar{X}-\bar{u}}\right)$ and $h\left(\bar{u}\right) = \exp\left(\frac{\bar{X}}{2\bar{X}-\bar{u}}\right)$ respectively.

For more detail see Diana et al. (2011) and Riaz et al. (2014).

Ranked set sampling (RSS)

The RSS procedure is described as in the following steps:

Step 1: Collect ν independent sets each of ν units.

Step 2: Array each set inside in ascending order by mean of the study variable or any closely related auxiliary variable. The ranking is done either by visual inspection or some quantitative measurements.

Step 3: Select the lowest order unit from the first set.

Step 4: Select the second lowest order unit from the second set and continue selecting units in this way until ν^{th} order statistic is selected from ν^{th} set.

This completes one cycle of an RSS procedure which can be repeated r times to obtain a sample of size $n = r\nu$. The selected units can be represented as $Y_{1(1)j}, Y_{2(2)j}, ..., Y_{i(i)j}, ..., Y_{\nu(\nu)j}$; $i = 1, 2, ..., \nu$, $j = 1, 2, ..., r$.

RSS under non-response

Bouza-Herrera (2013) has done a very comprehensive work on dealing with the missing observations under ranked set sampling. Here we discuss the following two major situations of dealing non-response under ranked set sampling.

Situation-I: When RSS is used at second attempt only.

Let we collect data at second attempt by method of RSS such a way that ν independent sets each of size ν are selected from the non-response group. The later procedure is followed step by step as discussed in Section 3 earlier. The estimate of sample mean from non-responding units based on $n_2' = r_2'\nu$ sampled units under RSS is given by,

$$\bar{y}'_{2rss} = \frac{1}{n_2'} \sum_{i=1}^{n_2'} \bar{y}'_{2(i)} \tag{12}$$

If the population is symmetrically distributed then $\bar{y}'_{2rss}$ is an unbiased estimator of the population mean with the following expected variance,

$$Var\left(\bar{y}'_{2rss}\right) = \frac{\sigma_2^2}{n_2'} - \frac{1}{n_2'^2} \sum_{i=1}^{n_2'} \Delta'^2_{2y(i)} \tag{13}$$

where

$\Delta'_{2y(i)} = \mu_{2y(i)} - \mu_{2y}$

Thus Eq (1) becomes,

$$\bar{y}^*_{rss} = w_1 \bar{y}_1 + w_2 \bar{y}'_{2rss} \tag{14}$$

$\bar{y}^*_{rss}$ is an unbiased estimator for which the variance is given by,

$$Var\left(\bar{y}^*_{rss}\right) = \frac{\sigma_y^2}{n} + \frac{W_2\left(K-1\right)\sigma_{2y}^2}{n} - W_2\left(\frac{K}{n\nu}\sum_{i=1}^{\nu}\Delta'^2_{2y(i)}\right) = V'_{yr} \tag{15}$$

similar result can be obtained for the auxiliary variable as,

$$Var\left(\bar{x}^*_{rss}\right) = \frac{\sigma_x^2}{n} + \frac{W_2\left(K-1\right)\sigma_{2x}^2}{n} - W_2\left(\frac{K}{n\nu}\sum_{i=1}^{\nu}\Delta'^2_{2x(i)}\right) = V'_{xr} \tag{16}$$

Situation-II: When RSS is used at both attempts (RSS on both groups).

It is extension of Situation-I in which we collect a sample from both groups by using RSS. The estimator of sample mean can be written as,

$$\bar{y}^{**}_{rss} = w_1 \bar{y}_{1rss} + w_2 \bar{y}'_{2rss} \tag{17}$$

where $\bar{y}_{1rss}$ is mean of sample based on n_1 units collected at first attempt, while $\bar{y}'_{2rss}$ is sample mean calculated from n_2' units collected at second attempt. $\bar{y}^{**}_{rss}$ is also an unbiased estimator which has the variance given as,

$$Var\left(\bar{y}^{**}_{rss}\right) = \frac{\sigma_y^2}{n} + \frac{W_2\left(K-1\right)}{n}\sigma_{y2}^2 - \frac{1}{r\nu^2}\left(\sum_{i=1}^{\nu}\Delta^2_{y[i]} + \left(K-1\right)\sum_{i=1}^{\nu}\Delta^2_{2y[i]}\right) = V''_{yr} \tag{18}$$

similar result can be obtained for the auxiliary variable as,

$$Var\left(\bar{x}^{**}_{rss}\right) = \frac{\sigma_x^2}{n} + \frac{W_2\left(K-1\right)}{n}\sigma_{x2}^2 - \frac{1}{r\nu^2}\left(\sum_{i=1}^{\nu}\Delta^2_{x[i]} + \left(K-1\right)\sum_{i=1}^{\nu}\Delta^2_{2y[i]}\right) = V''_{xr} \tag{19}$$

Note that the set size ν is kept constant while other notations are used as,

$n_1 = r_1\nu,\ \ n_2 = r_2\nu,\ \ n_2' = r_2'\nu,\ \ r = r_1 + r_2',\ \ n = r\nu,\ \ k = \frac{n_2}{n'_2} = \frac{r_2}{r'_2}$

Generalized class of Rao-regression type estimators under RSS in the presence of non-response

We extend the work of Rao (1991) and Diana et al. (2011) for the estimation of finite population mean when non-response occurs in surveys and RSS is used for the collection of data instead of SRS.

Our first suggested estimator is,

$$t_{r1} = q_1 \bar{y}^*_{rss} + q_2 \left(\bar{X} - \bar{x}^*_{rss} \right) \tag{20}$$

where q_1 and q_2 are constants, whose optimum values are used to minimize the error of estimate.

Equation (20) can be written as;

$$t_{r1} - \bar{Y} = \bar{Y} \left(q_1 - 1 \right) + q_1 \left(\bar{y}^*_{rss} - \bar{Y} \right) + q_2 \left(\bar{X} - \bar{x}^*_{rss} \right) \tag{21}$$

The error terms are defined as $\bar{v}^*_{rss} = \bar{y}^*_{rss} - \bar{Y}$ and $\bar{u}^*_{rss} = \bar{X} - \bar{x}^*_{rss}$, then it is easy to calculate that;

$$E\left(\bar{v}^*_{rss}\right) = E\left(\bar{u}^*_{rss}\right) = 0$$

and

$$E\big(\bar{v}^{*2}_{rss}\big) = V_{yr} = \begin{cases} V'_{yr}, & \text{if RSS is used on second attempt only} \\ V''_{yr}, & \text{if RSS is used at second attempt only} \end{cases}$$

$$E\big(\bar{u}^{*2}_{rss}\big) = V_{xr} = \begin{cases} V'_{xr}, & \text{if RSS is used on second attempt only} \\ V''_{xr}, & \text{if RSS is used at second attempt only} \end{cases}$$

The bias and MSE of t_r1, is given by,

$$Bias(t_{r1}) = \bar{Y} \left(q_1 - 1 \right)$$

$$MSE(t_{r1}) = \bar{Y}^2 (q_1 - 1)^2 + q_1^2 \bar{v}^{*2}_{rss} + q_2 \bar{u}^{*2}_{rss} - 2 q_1 q_2 \text{cov}\left(\bar{v}^*_{rss}, \bar{u}^*_{rss}\right)$$

or

$$MSE(t_{r1}) = \bar{Y}^2 \left(1 + q_1^2 - 2q_1\right) + q_1^2 V_{yr} + q_2 V_{xr} - 2 q_1 q_2 \rho_{yx} \sqrt{V_{yr} V_{xr}}$$

The optimum values of q_1 and q_2 with minimum MSE are given as

$$q_{1opt} = \frac{\bar{Y}^2 V_{xr}}{\bar{Y}^2 V_{xr} + V_{xr} V_{yr} - V^2_{yxr}}, \quad q_{2opt} = \frac{\bar{Y}^2 V_{yxr}}{\bar{Y}^2 V_{xr} + V_{xr} V_{yr} - V^2_{yxr}}$$

$$MSE(t_{r1})_{\min} = \frac{\bar{Y}^2 \left(V_{xr} V_{yr} - V^2_{yxr} \right)}{\bar{Y}^2 V_{xr} + V_{xr} V_{yr} - V^2_{yxr}} \tag{22}$$

it should be noted that the value of q_{1opt} is mathematically always a positive quantity, while the nature of sign for q_{2opt} is depending upon the correlation coefficient between Y and X.

Similarly, our second suggested general class of estimators is given as,

$$t_{ri} = \left(q_3 \bar{y}^*_{rss} + q_4 \bar{u}^*_{rss} \right) h\left(\bar{u}^*_{rss}\right) \tag{23}$$

where q_3 and q_4 are pre-determined constants and h is generic function of $\bar{u}^*_{rss} = \bar{X} - \bar{x}^*_{rss}$ that is satisfying the following mild conditions,

- Function h is bounded in the vicinity of zeros and is continous.
- Function h is independent of n, N and $(x_1, x_2, ..., x_N)$.

- Function h is thrice differentiable with bounded and continuous derivatives.

Equation (23) can be written as,

$$t_{ri} = \left(q_3 \bar{Y} + q_3 \bar{v}_{rss}^{*} + q_4 \bar{u}_{rss}^{*}\right) h\left(\bar{u}_{rss}^{*}\right)$$

Expanding $h\left(\bar{u}_{rss}^{*}\right)$ using Taylor series up to including terms i.e., $O_p\left(\bar{u}_{rss}^{*2}\right)$, the resulting expression can be written as,

$$\begin{aligned} t_{ri} = q_3 \bar{Y} \left[h\left(0\right) + h'\left(0\right) \bar{u}_{rss}^{*} + \frac{1}{2} h''\left(0\right) \bar{u}_{rss}^{*2}\right] + q_3 \bar{v}_{rss}^{*} \left[h\left(0\right) + h'\left(0\right) \bar{u}_{rss}^{*}\right] h\left(\bar{u}_{rss}^{*}\right) \\ + q_3 \bar{u}_{rss}^{*} \left[h\left(0\right) + h'\left(0\right) \bar{u}_{rss}^{*}\right] \end{aligned}$$

where $h(0)$ is a constant term, $h'(0)$ is first order partial derivative in zero and $h''(0)$ is second order partial derivative in zero. For simplicity we write $h(0) = a$, $h'(0) = b$ and $h''(0) = c$. Thus,

$$t_{ri} = q_3 \bar{Y} \left[a + b \bar{u}_{rss}^{*} + \frac{1}{2} c \bar{u}_{rss}^{*2}\right] + q_3 \bar{v}_{rss}^{*} \left[a + b \bar{u}_{rss}^{*}\right] h\left(\bar{u}_{rss}^{*}\right) + q_3 \bar{u}_{rss}^{*} \left[a + b \bar{u}_{rss}^{*}\right]$$

or

$$t_{ri} - \bar{Y} = \bar{Y}\left(a q_3 - 1\right) + \left(a w_1 + b w_0 \bar{Y}\right) \bar{u}_{rss}^{*} + a w_0 \bar{v}_{rss}^{*} + (c w_0 \bar{Y} + b w_1) \bar{u}_{rss}^{*2} + b w_0 \bar{u}_{rss}^{*} \bar{v}_{rss}^{*} \tag{24}$$

The bias and MSE of t_{ri} , are given by,

$$Bias\left(t_{ri}\right) = \bar{Y}\left(a q_3 - 1\right) + \left(c w_0 \bar{Y} + b w_1\right) V_{xr} + b w_0 V_{yxr}$$

$$\begin{aligned} MSE\left(t_{ri}\right) = \bar{Y}^2 (a q_1 - 1)^2 + \left[\left(a q_2 + b q_1 \bar{Y}\right)^2 + 2\bar{Y}\left(a q_1 - 1\right)\left(c q_1 \bar{Y} + b q_2\right)\right] V_{xr} \\ + a q_1^2 V_{yr} + \left[2 a q_1 \left(a q_2 + b q_1 \bar{Y}\right) + 2 b q_1 \bar{Y}\left(a q_1 - 1\right)\right] \rho_{yx} \sqrt{V_{yr} V_{xr}} \end{aligned} \tag{25}$$

The optimum values of q_3 and q_4 are calculated as,

$$q_{3opt} = \frac{\bar{Y}^2 V_{xr}\left(a^2 - 2b^2 V_{xr} + ac V_{xr}\right)}{a^3\left(\bar{Y}^2 V_{xr} + V_{xr} V_{yr} - \rho_{yx} V_{xr} V_{yr}\right) + a \bar{Y}^2\left(2ac - 3b^2\right) V_{xr}^2}$$

$$q_{4opt} = \frac{\bar{Y}\left[P + b^3 \bar{Y}^2 V_{xr}^2 + \left(a^3 \bar{Y} - 2ab^2 \bar{Y} V_{xr}\right) \rho_{yx} \sqrt{V_{yr} V_{xr}}\right]}{V_{xr}\left[a^4\left(\bar{Y}^2 + V_{yr} - \rho_{yx} V_{yr}\right) + a^2 \bar{Y}^2\left(2ac - 3b^2\right) V_{xr}\right]}$$

the minimum MSE of t_{ri} is given as,

$$MSE(t_{ri})_{\min} = \frac{\bar{Y}^2\left[a^4 V_{yr}^3\left(1 - \rho_{yx}\right) + b^2 \bar{Y}^2 V_{xr}^3\left(2ac - b^2\right) - Q\right]}{a^2\left[a^2\left(\bar{Y}^2 + V_{yr} - \rho_{yx} V_{yr}\right) + \bar{Y}^2 V_{xr}\left(2ac - 3b^2\right)\right]} \tag{26}$$

where
$P = a^2 V_{xr}\left[c \bar{Y} \rho_{yx} V_{yr} V_{yr} - b\left(\bar{Y}^2 - V_{yr} + \rho_{yx} V_{yr}\right)\right]$,
$Q = a^2 V_{xr}\left\{b^2 V_{yr}\left(1 - \rho_{yx}\right) + c^2 \bar{Y}^2 V_{xr}\right\}$.

Choice of function h

The proposed class of estimators is determined by the function which can theoretically and practically take into account a variety of options but in our study, we discuss the following two well-known functions.

The ratio function

We consider Singh (1982) and Kadilar & Cingi (2004) estimators as a choice for ratio function h.

$$h\left(\bar{u}^*_{rss}\right) = \left(\frac{\bar{X}}{\bar{X} - \bar{u}^*_{rss}}\right)^{\tau} \tag{27}$$

where τ is a constant, and

$h\left(0\right) = a = 1,$

$h'\left(0\right) = b = -\tau$

$\frac{1}{2}h''\left(0\right) = c = \frac{\tau(\tau+1)}{2}.$

Remarks

1. If we consider $\tau = 0$, then $\{a, b, c\} = \{1, 0, 0\}$ and the proposed estimator t_{ri} is converted to proposed estimator t_{r1} as,

$$t_{r1} = q_1 \bar{y}^*_{rss} + q_2\left(\bar{X} - \bar{x}^*_{rss}\right) \tag{28}$$

2. If we consider $\tau = 1$, then $\{a, b, c\} = \{1, -1, 1\}$ and the function h is converted to Ray and Singh (1981) estimator. Thus the proposed estimator becomes,

$$t_{r2} = \left(q_3 \bar{y}^*_{rss} + q_4 \bar{u}^*_{rss}\right)\left(\frac{\bar{X}}{\bar{X} - \bar{u}^*_{rss}}\right) \tag{29}$$

3. If we consider $\tau = -1$, then $\{a, b, c\} = \{1, 1, 0\}$. Hence the proposed estimator is converted to the product type estimator which is used when the correlation coefficient between study variable and auxiliary variable is negative i.e.,

$$t_{rp} = \left(q_3 \bar{y}^*_{rss} + q_4 \bar{u}^*_{rss}\right)\left(\frac{\bar{X} - \bar{u}^*_{rss}}{\bar{X}}\right) \tag{30}$$

The exponential function

Now we consider the exponential function proposed by Bahl & Tuteja (1991) as a possible choice of function h i.e.,

$$h\left(\bar{u}^*_{rss}\right) = \exp\left(\frac{\bar{u}^*_{rss}}{2\bar{X} - \bar{u}^*_{rss}}\right) \tag{31}$$

Then proposed class of estimator takes the following form,

$$t_{r3} = \left(q_3 \bar{y}^*_{rss} + q_4 \bar{u}^*_{rss}\right)\exp\left(\frac{\bar{u}^*_{rss}}{2\bar{X} - \bar{u}^*_{rss}}\right) \tag{32}$$

By expanding $h\left(\bar{u}^*_{rss}\right)$ and using Taylor's series, we get,

$h\left(0\right) = a = 1$

$h'\left(0\right) = b = -\frac{1}{2}$

$\frac{1}{2}h''\left(0\right) = c = \frac{3}{8}$

By using the values of constants a, b and c, we can easily calculate the corresponding optimum values of q_3 and q_4 with minimum MSE of estimators by using Eq (26), for the two choices of function h discussed above .

Efficiency comparison

The gain in precision of the proposed class of estimators is completely relying on the precision gained in the estimation by using RSS instead of SRS sampling method. So, it is reasonable to compare the variances of sample mean by using the two competing sampling strategies. Consider the variance of $\bar{y}_{srs}$

$$Var\left(\bar{y}_{rss}\right) = \frac{\sigma_y^2}{n} - \frac{1}{mn}\sum_{i=1}^{m}\left(\mu_{y(i)} - \mu_y\right)^2 \tag{33}$$

In the above expression the term $\frac{\sigma_y^2}{n}$ is same as the variance of $\bar{y}_{srs}$, which indicates that the RSS procedure will produce more precise estimates than the SRS if the inequality $\mu_{y(i)} \neq \mu_y$ holds. This also leads to a certainty that if we use the RSS procedure for collecting data at both attempts, then the proposed class of estimators will be more precise than the estimators discussed under situation-I where we collect data through RSS only at second attempt.

Simulation study

The simulation study is carried out in the context of assumptions made by Hansen & Hurwitz (1946). We generated the auxiliary variable for the two groups as $X_j \sim Normal\left(N_j, \mu_{yj}, \sigma_{yj}\right)$; $j = 1, 2$. The corresponding study variable is produced by using the relationship $Y_j = \rho_{yx}X_j + e_j\sqrt{1-\rho_{yx}^2}$, where $e_j \sim Normal\left(N_j, 0, 1\right)$ and ρ_{yx} is the coefficient of correlation between Y and X. A sample of size $n = (n_1 + n_2')$ is selected from population by using the procedure of SRS and RSS and sample mean are calculated for the study and auxiliary variable under Situation-I and Situation-II. Then the sample mean for competing and proposed estimators is estimated. This procedure is repeated 20,000 times to calculate the MSE and RE of the estimators using the following formula,

$$MSE(t) = \frac{1}{20,000}\sum_{j=1}^{20,000}\left(t - \mu_y\right)^2 \tag{34}$$

$$RE\left(t_{ri}\right) = \frac{MSE\left(t_{si}\right)}{MSE(t_{ri})}, \quad i = 1, 2, 3 \tag{35}$$

where t represents an estimator under consideration. The results are given in tables 1-6.

Table 1. RE of proposed class of estimators for Situation-I when $K = 2$.

r_2'	Est	$\rho = 0.10$			$\rho = 0.50$			$\rho = 0.90$		
		$\nu = 3$	$\nu = 4$	$\nu = 5$	$\nu = 3$	$\nu = 4$	$\nu = 5$	$\nu = 3$	$\nu = 4$	$\nu = 5$
1	t_{r1}	1.510	1.520	1.563	1.502	1.516	1.530	1.591	1.603	1.620
	t_{r2}	5.151	7.094	8.595	6.825	10.10	13.47	6.896	10.23	13.78
	t_{r3}	3.370	4.230	4.773	6.924	10.12	13.20	7.310	10.80	14.45
2	t_{r1}	1.500	1.514	1.562	1.580	1.580	1.610	1.570	1.583	1.680
	t_{r2}	9.182	12.84	15.55	13.12	19.96	21.83	13.31	18.30	21.44
	t_{r3}	5.350	6.870	8.020	13.06	19.71	21.17	13.98	19.28	23.60
3	t_{r1}	1.572	1.573	1.540	1.610	1.701	1.720	1.690	1.721	1.740
	t_{r2}	12.55	14.98	16.12	14.92	20.37	25.88	19.29	22.98	26.77
	t_{r3}	6.920	9.192	10.75	14.69	21.77	27.83	15.19	22.31	27.48

Table 2. RE of proposed class of estimators for Situation-I when $K = 3$.

r_2'	Est	$\rho = 0.10$			$\rho = 0.50$			$\rho = 0.90$		
		$\nu = 3$	$\nu = 4$	$\nu = 5$	$\nu = 3$	$\nu = 4$	$\nu = 5$	$\nu = 3$	$\nu = 4$	$\nu = 5$
1	t_{r1}	1.260	1.331	1.322	1.360	1.420	1.410	1.360	1.410	1.430
	t_{r2}	5.061	7.080	7.631	5.710	8.350	11.77	5.810	9.600	12.13
	t_{r3}	3.273	4.120	4.051	5.760	11.19	11.40	7.250	10.19	13.91
2	t_{r1}	1.341	1.321	1.360	1.390	1.400	1.460	1.400	1.420	1.460
	t_{r2}	8.962	11.92	14.65	12.50	16.32	21.18	12.79	14.70	16.86
	t_{r3}	5.053	6.030	7.460	10.36	15.99	20.38	11.49	16.78	21.26
3	t_{r1}	1.350	1.351	1.370	1.450	1.440	1.520	1.510	1.540	1.520
	t_{r2}	11.63	12.99	14.74	12.18	16.60	21.65	17.48	20.22	21.29
	t_{r3}	6.601	9.140	10.04	12.06	20.99	24.81	13.55	21.70	22.42

Table 3. RE of proposed class of estimators for Situation-I when $K = 4$

r_2'	Est	$\rho = 0.10$			$\rho = 0.50$			$\rho = 0.90$		
		$\nu = 3$	$\nu = 4$	$\nu = 5$	$\nu = 3$	$\nu = 4$	$\nu = 5$	$\nu = 3$	$\nu = 4$	$\nu = 5$
1	t_{r1}	1.103	1.101	1.133	1.113	1.140	1.172	1.124	1.172	1.195
	t_{r2}	4.781	6.951	6.958	5.387	9.241	9.670	5.520	9.522	12.12
	t_{r3}	3.240	4.103	4.031	4.358	9.960	9.102	6.974	11.17	12.93
2	t_{r1}	1.153	1.161	1.160	1.183	1.192	1.210	1.180	1.195	1.251
	t_{r2}	8.152	10.08	12.56	11.18	15.98	16.25	10.47	11.58	13.08
	t_{r3}	4.311	5.682	6.831	9.970	12.22	14.98	10.24	14.73	18.69
3	t_{r2}	1.201	1.212	1.242	1.242	1.251	1.302	1.274	1.345	1.412
	t_{r2}	10.77	11.08	13.04	11.43	13.36	15.04	15.78	18.03	20.38
	t_{r3}	5.123	8.570	9.621	11.10	16.50	22.73	16.99	17.70	22.72

Table 4. RE of proposed class of estimators for Situation-II when $K = 2$

(r_1, r_2')	Est	$\rho = 0.10$			$\rho = 0.50$			$\rho = 0.90$		
		$\nu = 3$	$\nu = 4$	$\nu = 5$	$\nu = 3$	$\nu = 4$	$\nu = 5$	$\nu = 3$	$\nu = 4$	$\nu = 5$
(3, 1)	t_{r1}	1.932	1.856	1.940	2.022	2.011	2.088	2.003	1.992	2.040
	t_{r2}	5.487	6.353	7.450	8.332	11.18	14.50	8.470	11.43	15.11
	t_{r3}	3.549	3.760	4.232	8.051	10.57	13.27	8.732	11.75	15.42
(3, 2)	t_{r1}	1.809	1.830	1.670	2.010	2.006	1.962	1.990	1.950	1.920
	t_{r2}	7.152	8.882	12.01	11.74	16.56	22.35	11.96	17.04	23.15
	t_{r3}	4.185	4.846	5.762	11.22	15.39	20.28	12.28	17.39	23.54
(3, 3)	t_{r1}	1.760	1.605	1.650	1.982	1.935	2.007	1.940	1.903	1.930
	t_{r2}	8.772	10.26	12.34	14.58	22.14	29.18	14.88	22.72	30.27
	t_{r3}	4.880	5.122	5.878	13.85	20.46	26.39	15.22	23.14	30.63

Table 5. RE of proposed class of estimators for Situation-II when $K = 3$

(r_1, r_2')	Est	$\rho = 0.10$			$\rho = 0.50$			$\rho = 0.90$		
		$\nu = 3$	$\nu = 4$	$\nu = 5$	$\nu = 3$	$\nu = 4$	$\nu = 5$	$\nu = 3$	$\nu = 4$	$\nu = 5$
(3, 1)	t_{r1}	1.773	1.844	1.550	1.940	2.184	1.794	1.930	2.186	1.790
	t_{r2}	4.511	5.926	5.584	6.805	10.99	11.16	6.911	11.33	11.49
	t_{r3}	2.970	3.499	3.168	6.528	10.17	10.23	7.036	11.47	11.65
(3, 2)	t_{r1}	1.613	1.548	1.430	1.974	1.993	1.930	1.980	2.007	1.950
	t_{r2}	5.423	6.466	6.900	8.900	12.34	15.58	9.082	12.56	15.92
	t_{r3}	3.270	3.566	3.563	8.468	11.58	14.31	9.220	12.75	16.10
(3, 3)	t_{r1}	1.493	1.406	1.333	1.914	1.920	1.840	1.916	1.940	1.868
	t_{r2}	6.080	7.110	7.610	10.49	14.54	18.85	10.63	14.67	19.00
	t_{r3}	3.463	3.673	3.734	9.996	13.68	17.46	10.80	14.88	19.24

Table 6. RE of proposed class of estimators for Situation-II when $K = 4$

(r_1, r_2')	Est	$\rho = 0.10$			$\rho = 0.50$			$\rho = 0.90$		
		$\nu = 3$	$\nu = 4$	$\nu = 5$	$\nu = 3$	$\nu = 4$	$\nu = 5$	$\nu = 3$	$\nu = 4$	$\nu = 5$
(3, 1)	t_{r1}	1.690	1.547	1.535	1.995	1.902	1.949	2.009	1.935	1.970
	t_{r2}	4.458	4.748	4.990	7.738	9.548	11.87	7.838	9.630	12.18
	t_{r3}	2.833	2.812	2.864	7.365	8.977	10.82	7.942	9.794	12.30
(3, 2)	t_{r1}	1.479	1.349	1.282	1.934	1.870	1.790	1.980	1.954	1.872
	t_{r2}	4.461	4.970	5.070	8.536	11.96	14.72	8.657	12.00	14.88
	t_{r3}	2.733	2.765	2.742	8.095	11.22	13.46	8.762	12.20	15.07
(3, 3)	t_{r1}	1.360	1.213	1.219	1.932	1.706	1.793	2.004	1.797	1.901
	t_{r2}	4.739	4.961	5.168	9.860	14.03	17.38	9.887	14.05	17.35
	t_{r3}	2.755	2.670	2.730	9.447	13.03	15.98	10.03	14.27	17.61

simulation results in Table 1-6 shows that the relative efficiency of proposed estimators increases as the value of correlation coefficient increase. the relative efficiency also increases with the increase in overall sample size n, while relative efficiency is observed highest when moderate set size $\nu = 4$ is used. However, the relative efficiency decreases when the value of non-response rate K is increased.

1 Conclusions

From the simulation results it is observed that the proposed class of estimators is provides more precise results for estimating population mean when non-response occurs in surveys. Furthermore, relative efficiency of proposed class of estimators is higher when RSS is used to collect data at both attempts than using RSS for data collection only on second attempt. So, it is suggested to use the proposed class of estimators under RSS sampling technique. The use of RSS on both attempts of data collection is recommended for the more gain in efficiency of estimates.

Acknowledgments

I would like to express my gratitude to my supervisor, Javid shabbir, who guided me throughout this paper.

References

Abu-Dayyeh, W. A., Ahmed, M. S., Ahmed, R. & Muttlak, H. A. (2003), 'Some estimators of a finite population mean using auxiliary information', *Applied Mathematics and Computation* **139**(2-3), 287–298.

Al-Omari, A. & Bouza, C. (2015), 'Ratio estimators of the population mean with missing values using ranked set sampling', *Environmetrics* **26**(2), 67–76.

Bahl, S. & Tuteja, R. (1991), 'Ratio and product type exponential estimators', *Journal of information and optimization sciences* **12**(1), 159–164.

Bouza-Herrera, C. N. (2013), *Handling missing data in ranked set sampling*, Springer.

Cochran, W. G. (1977), *Sampling techniques*, John Wiley & Sons.

Diana, G., Giordan, M. & Perri, P. F. (2011), 'An improved class of estimators for the population mean', *Statistical Methods & Applications* **20**(2), 123–140.

Fatima, M., Shahbaz, S. H., Hanif, M. & Shahbaz, M. Q. (2022), 'A modified regression-cum-ratio estimator for finite population mean in presence of nonresponse using ranked set sampling', *AIMS Mathematics* **7**(4), 6478–6488.

Hansen, M. H. & Hurwitz, W. N. (1946), 'The problem of non-response in sample surveys', *Journal of the American Statistical Association* **41**(236), 517–529.

Kadilar, C. & Cingi, H. (2004), 'Ratio estimators in simple random sampling', *Applied mathematics and computation* **151**(3), 893–902.

Kadilar, C. & Cingi, H. (2005), 'A new estimator using two auxiliary variables', *Applied Mathematics and Computation* **162**(2), 901–908.

Khare, B. & Srivastava, S. (1997), 'Transformed ratio type estimators for the population mean in the presence of nonresponse', *Communications in Statistics-Theory and Methods* **26**(7), 1779–1791.

Lynne Stokes, S. (1977), 'Ranked set sampling with concomitant variables', *Communications in Statistics-Theory and Methods* **6**(12), 1207–1211.

Malik, S. & Singh, R. (2013), 'An improved estimator using two auxiliary attributes', *Applied mathematics and computation* **219**(23), 10983–10986.

McIntyre, G. (1952), 'A method for unbiased selective sampling, using ranked sets', *Australian journal of agricultural research* **3**(4), 385–390.

Muneer, S., Shabbir, J. & Khalil, A. (2017), 'Estimation of finite population mean in simple random sampling and stratified random sampling using two auxiliary variables', *Communications in Statistics-Theory and Methods* **46**(5), 2181–2192.

Rao (1987), 'Ratio and regression estimates with sub sampling the non-respondents', *International Statistical Association Meeting* (2), 2–16.

Rao, P. (1986), 'Ratio estimation with sub sampling the non-respondents', *Survey methodology* **12**(2), 217–230.

Rao, T. (1991), 'On certail methods of improving ration and regression estimators', *Communications in Statistics-Theory and Methods* **20**(10), 3325–3340.

Rehman, S. A. & Shabbir, J. (2021), 'On the improvement of paired ranked set sampling to estimate population mean', *Statistics in Transition New Series* **22**(3).

Riaz, S., Diana, G. & Shabbir, J. (2014), 'Improved classes of estimators for population mean in presence of non-response.', *Pakistan Journal of Statistics* **30**(1).

Singh, H. P., Tailor, R. & Singh, S. (2014), 'General procedure for estimating the population mean using ranked set sampling', *Journal of Statistical Computation and Simulation* **84**(5), 931–945.

Singh, R. K. (1982), 'On estimating ratio and product of population parameters', *Calcutta Statistical Association Bulletin* **31**(1-2), 69–76.

Singh, R. & Sharma, P. (2015), 'A class of exponential ratio estimators of finite population mean using two auxiliary variables', *Pakistan Journal of Statistics and Operation Research* pp. 221–229.

Takahasi, K. & Wakimoto, K. (1968), 'On unbiased estimates of the population mean based on the sample stratified by means of ordering', *Annals of the institute of statistical mathematics* **20**(1), 1–31.
