# Supplementary material for: An efficient class of estimators for finite population mean in the presence of non-response under ranked set sampling (RSS)
Source: PLoS One. 2022 Dec 16;17(12):e0277232. doi: 10.1371/journal.pone.0277232 (PMC9757593; doi:10.1371/journal.pone.0277232)
Supplement: Attachment — Submitted filename: Report for the paper.pdf [file pone.0277232.s004.pdf]

Paper ID: PONE-D-22-15506R1

Report of the paper **“**An efficient class of estimators for finite population mean in the presence of non-response under ranked set sampling (RSS)**”** Submitted to PLOS ONE

<u>Referee’s Comments</u>

In this paper, authors have suggested a class of Rao-regression type estimators and ranked set sampling (RSS) scheme is used to collect data from different situations non-response response Expressions for bias and mean square error of the estimators are obtained up to first order of approximation and comprehensive simulation study is carried out to observe the performances of the proposed estimators under non-response. Authors have incorporated all the comments raised by the previous reviewer and I agreed with the response. The idea/concept of the paper is interesting and can be published after some corrections. Some suggestions are as follows:

1) I would like to suggest the authors to make an Appendix to post all codes related to this paper in the next submission.
2) Add these references in the reference section as well in the text section
    a. Khalid (2019). Effective Estimation Strategy of Population Variance in Two-Phase Successive Sampling Under Random Non-response. *J Stat Theory Pract* **13,** 4 https://doi.org/10.1007/s42519-018-0010-y
    b. Mohd Khalid. "Exponential chain dual to ratio and regression type estimators of population mean in two-phase sampling." *Statistica* 75.4 (2015): 379-389.
    c. Khalid, Mohd. "Some imputation methods to deal with the issue of missing data problems due to random non-response in two-occasion successive sampling." *Communications in Statistics-Simulation and Computation* (2020): 1-21.
