# Supplementary material for: An efficient class of estimators for finite population mean in the presence of non-response under ranked set sampling (RSS)
Source: PLoS One. 2022 Dec 16;17(12):e0277232. doi: 10.1371/journal.pone.0277232 (PMC9757593; doi:10.1371/journal.pone.0277232)
Supplement: Attachment — Submitted filename: Response to the reviewers.docx [file pone.0277232.s002.docx]

**PONE-D-22-15506**

An efficient class of estimators for the population mean in the presence of non-response under Ranked Set Sampling

We are thankful to the editor for their valuable suggestions. We have revised and changed the following points in our manuscript as pointed out by the editors.

1. *“As follows from the first sentence, the authors claim that estimation of population parameters are unreliable if the distribution of the general population is asymmetric. This is a false statement. Many distributions we deal with are asymmetric, like exponential, gamma, etc. I’ve never heard that parameters estimators are unreliable. What the authors mean?”*

**Ans.** We have removed the sentence from the manuscript and the updated sentence read as follows:

*A survey can be conducted using a variety of sampling techniques depending upon the situation and structure of data. Simple random sampling (SRS) is an easy and unbiased data collection method, but in situations where the overall population is dispersed and diverse, it requires a larger sample for a given margin of accuracy.*

2. *The second sentence is about response and non-response. Do the authors talk about surveys? What is “pattern?” Do they mean that people who do not response constitute a different distribution? Can’t we treat them as missing observations?*

**Ans. Though, it has been observed that the non-responding units can follow any other distribution other than followed by responding units. For example, In social sciences, non-respondents can represent a whole group (Ethnic, religious, political), in which elements are similar to each other and different from responding units (i.e., its pattern or distribution can be changed from responding units). In biological sciences, the non-respondents can represent a specific bacteria, virus, or disease. In animal sciences it can be a specific species or breed, in chemical sciences it can be a different chemical.**

**Since we did not find a significant work which could be referenced for this point, so we decided to change this sentence too with the following sentence which is linked to the previous sentence.**

*On the other hand, estimation of population parameter becomes more problematic if non-response occurs in the data collected from such a scattered population. Non-response is an inability to collect data from one or more units of population units selected in a sample frame.*

3. *English is bad in the third sentence of Section 1.1: “This appeals that in the existence…” I simply do not understand what the authors wanted to say.*

**Ans. We agreed to the editor’s objection and decided to replace the sentence with the following,**

*Most of the time, we have to generalize the survey results by using a smaller sample which demands that an appropriate sampling method should be employed to* ***obtain*** *a sample that could represent the population well enough.*

4. *The problem set up in Section 2 makes little sense. How can we divide the general population Ω into response group Ω1 and non-response group Ω2 before surveys have been conducted? We can split a sample into response and non-response groups not a general population. The language is so sloppy that the reader even does not understand the problem set up.*

**Ans. The paragraph is removed, and new paragraph reads as follows,**

*We consider the naive model of Hansen and Hurwitz (1946). According to which, we draw a sample s of size* $n_{s}$ *by using SRSWOR method from a finite population* $\Omega$ *of size* $N$*. Let* $n_{1}$ *units respond to the survey at first attempt while* $n_{2}\left(=n-n_{1}\right)$ *units do not respond. Special efforts are made to approach the non-responding units and a part of them* $(n'_{2}=n_{2}/k;\text{ }k>1)$ *is included in the sample. Thus, we get a final sample of size* $n=n_{1}+n'_{2}$ *for estimation purpose. This allows the entire population is to be separated into two complementary groups called response and non-response group.*

*Let* $\left(Y_{ji},\text{ }X_{ji}\right)$*;* $j=1,2$ *shows population units of the study variable* $\left(Y\right)$ *and the auxiliary variable* $\left(X\right)$ *in two groups. Our goal is to estimate the population mean* $\left(\bar{Y}\right)$ *of the study variable. Hansen and Hurwitz (1946) suggested the following unbiased estimator of the population mean* $\bar{Y}$ *when non-response occurs on* $Y.$

$\bar{y}_{srs}^{*}=w_{1}\bar{y}_{1}+w_{2}\bar{y}'_{2}$ (1)
