# Supplementary material for: An efficient class of estimators for finite population mean in the presence of non-response under ranked set sampling (RSS)
Source: PLoS One. 2022 Dec 16;17(12):e0277232. doi: 10.1371/journal.pone.0277232 (PMC9757593; doi:10.1371/journal.pone.0277232)
Supplement: Attachment — Submitted filename: Cover Letter.docx [file pone.0277232.s005.docx]

***To***

***The Editor in Chief***

**PLOS ONE**

***Dear Professor,***

With reference to e-mail dated on September 7-2022, I received the paper entitled **“**An efficient class of estimators for finite population mean in the presence of non-response under ranked set sampling (RSS)**”** Submitted to **PLOS ONE with the** PONE-D-22-15506R1**.** I have gone through the paper and found that authors have proposed interesting work. The review report is attached at the appropriate place.

I hope this will help the editor for taking the decision about manuscript.

*Thanking you*

*With regards*

*Referee*
