# Supplementary material for: An efficient class of estimators for finite population mean in the presence of non-response under ranked set sampling (RSS)
Source: PLoS One. 2022 Dec 16;17(12):e0277232. doi: 10.1371/journal.pone.0277232 (PMC9757593; doi:10.1371/journal.pone.0277232)
Supplement: Attachment — Submitted filename: Response to Reviewers.docx [file pone.0277232.s006.docx]

**Response to Reviewers**

**Dear Editor,**

Both authors are very thankful to the editor and all the reviewers for their precious time in reviewing our paper and providing valuable comments. It was your valuable comments, pointed issues, and insightful suggestions that led to possible improvements in the current version. The authors have carefully considered all the comments from reviewers, and addressed the issues pointed out in the manuscript with possible rectifications correspondingly. We hope the manuscript, after careful revisions, meets your high standards. The authors welcome any further constructive comments if any.

Below we provide the point-by-point responses, corrections, and clarifications. The comments of reviewers are highlighted in **red**, while the corresponding modifications and corrections are highlighted in **blue**.

| **Syed Abdul Rehman** | **Prof. Dr. Javid Shabbir** |
| --- | --- |
| Corresponding author | Co-author |
| BUITEMS, Quetta, Pakistan | University of Wah, Rawalpindi, Pakistan |
| rehmanbukhari725@gmail.com | javidshabbir@gmai.com |

**Reviewer #1:**

1. Authors need to update the introduction by incorporating the latest references regarding L-moments use.

**AUTHOR’S RESPONSE:**

Suggestion of the reviewer is accepted, and the introduction is updated by including the latest references regarding L-moments as given below:

In such case, researchers tend to employee robust methods for survey designing and parameter estimation. For example, some writers have proposed estimators of population mean based on L-moments theory, which is robust to outliers and less vulnerable to the effects of sample volatility. The L-moments theory includes the use of order statistics, and it leads to new procedures for estimating population parameters. Computation of the first few sample L-moments and their ratios provides a useful review of the location, shape, and dispersion for the population from which the sample was drawn. In this regard, the work of Coe (2019), Shahzad et al. (2021), Anas et al. (2021), Anas et al. (2022), and Lone et al. (2021) can be seen.

The corresponding references added in the manuscript are:

Anas, M. M., Huang, Z., Alilah, D. A., Shafqat, A., & Hussain, S. (2021). Mean estimators using robust quantile regression and L-moments’ characteristics for complete and partial auxiliary information. *Mathematical Problems in Engineering*, *2021*.

Shahzad, U., Ahmad, I., Almanjahie, I., et al. (2021). A new class of L-moments based calibration variance Estimators. Computers Materials and Continua, 66(3): 3013–3028. doi:10.32604/cmc.2021.014101.

Anas, M. M., Huang, Z., Shahzad, U., Zaman, T., & Shahzadi, S. (2022). Compromised imputation based mean estimators using robust quantile regression. *Communications in Statistics-Theory and Methods*, 1-16.

Lone, S. A., Subzar, M., & Sharma, A. (2021). Enhanced estimators of population variance with the use of supplementary information in survey sampling. *Mathematical Problems in Engineering*, *2021*.

2. Extend results interpretation.

**AUTHOR’S RESPONSE:**

Both authors opted to take the reviewer's advice, and the interpretations were extended carefully. The reviewer's advice was really helpful. Below is the Interpretation, before and after the change.

| **Before correction** | **After correction** |
| --- | --- |
| simulation results in Table 1-6 shows that the relative efficiency of proposed estimators increases as the value of correlation coefficient increase. the relative efficiency also increases with the increase in overall sample size n, while relative efficiency is observed highest when moderate set size ν = 4 is used. However, the relative efficiency decreases when the value of non-response rate K is increased. | Simulation results in Tables 1-6 show that the RE of the proposed class of estimators under RSS is higher than its competing estimators under SRS for estimating population mean. It is also observed that the RE increases when the correlation coefficient between *Y* and *X* is increased. The RE also increases by increasing the overall sample size *n*, while it decreases when the value of non-response rate *k* is increased. We also observed that the RE is comparatively higher for a moderate set size *v*, i.e., *v=4*. Furthermore, the RE is higher for Situation-I when compared to Situation-II of sampling selection under RSS procedure for the same sample size. |

3. Conclusion should be precise.

**AUTHOR’S RESPONSE:**

Conclusions are revised and written precisely. The conclusion before and after revision is given below. Also, both authors thank reviewer for advising this valuable suggestion.

| **Before correction** | **After correction** |
| --- | --- |
| From the simulation results it is observed that the proposed class of estimators is provides more precise results for estimating population mean when non-response occurs in surveys. Furthermore, relative efficiency of proposed class of estimators is higher when RSS is used to collect data at both attempts than using RSS for data collection only on second attempt. So, it is suggested to use the proposed class of estimators under RSS sampling technique. The use of RSS on both attempts of data collection is recommended for the more gain in efficiency of estimates. | In this paper, we proposed a generalized class of Rao-regression type estimators for finite population mean under non-response when RSS is used to collect the data at the second attempt and at both attempts. Expressions for bias and MSE of the proposed class of estimators were derived up to the first order of approximation. A brief simulation study was conducted to determine the RE of the proposed class of estimators for different values of non-response rate *k*, set size *v*, and overall sample size *n*.  Based on simulation results, we suggest using the proposed class of estimators under RSS for more precise estimation of the finite population mean when non-response occurs in the data. Additionally, we encourage using the RSS technique for data collection in both attempts since it provides more precise estimates. |

**Reviewer #2:**

Estimation of unknown mean under RSS framework and in situation of nonresponse is discussed. Some estimators are proposed, and their properties are studied. A simulation study is carried out in support of theoretical study.
Line 206- estimators is provides...some other language issues are there, read whole manuscript minutely.

**AUTHOR’S RESPONSE:**

We revised the manuscript thoroughly and corrected all the grammatical flaws carefully with the help of academic writer. Furthermore, the pointed sentence by reviewer is from conclusion section, which as a whole section is revised and corrected as given below,

| **Before correction** | **After correction** |
| --- | --- |
| From the simulation results it is observed that the proposed class of estimators is provides more precise results for estimating population mean when non-response occurs in surveys. Furthermore, relative efficiency of proposed class of estimators is higher when RSS is used to collect data at both attempts than using RSS for data collection only on second attempt. So, it is suggested to use the proposed class of estimators under RSS sampling technique. The use of RSS on both attempts of data collection is recommended for the more gain in efficiency of estimates. | In this paper, we proposed a generalized class of Rao-regression type estimators for finite population mean under non-response when RSS is used to collect the data at the second attempt and at both attempts. Expressions for bias and MSE of the proposed class of estimators were derived up to the first order of approximation. A brief simulation study was conducted to determine the RE of the proposed class of estimators for different values of non-response rate *k*, set size *v*, and overall sample size *n*.  Based on simulation results, we suggest using the proposed class of estimators under RSS for more precise estimation of the finite population mean when non-response occurs in the data. Additionally, we encourage using the RSS technique for data collection in both attempts since it provides more precise estimates. |

**Reviewer #3:**

1) In the simulation study, I am not if results remain unchanged for negative values of rho, e.g. for rho=+/-0.5. Could authors comment on this? If the answer is negative, the negative values can be considered in the study as well.

**AUTHOR’S RESPONSE:**

In estimation theory, a well-known fact is that we use a ratio estimator when the correlation coefficient is positive between the study variable and the auxiliary variable. In contrast, When the correlation coefficient is negative, the equivalent alternative is the product estimator which is well understood concept.

In our study, as a choice of *h* (of proposed estimator), we have considered the two functions (ratio and exponential) that are used when the correlation coefficient is positive between *Y* and *X*. In other words, our study is limited to only ratio-type functions of the auxiliary variables that are used when *Y* and *X* are positively correlated. (Screenshots from manuscript are given below).

and

These two functions can be converted to product-type estimators, which are used when the correlation between the two variables is negative, but that is beyond the scope of our study. Even if we include them in our study, it will require at least 6 more tables of simulation study, which makes the manuscript overexplained. So, both authors decided to keep the manuscript within the scope of the study.

However, any further suggestion from reviewer in this regard will be welcomed.

For the reviewer’s interest, here we explain the procedure RSS when the two variables are negatively correlated. In addition, simulation results are also given when *k=3*.

**RSS Procedure for negatively correlated variables**

Let we have to select *v* units in the sample by the method of RSS, and the auxiliary variable is negatively correlated with the study variable. Then we draw *v* independent sets, each of size *v* from the population and array the units in each set by descending order of the auxiliary variable. Further procedure is the same as given in the manuscript in the Section of “**RSS procedure**”.

It is worth noting that under this method, the simulation study provides less or more the same results as in the case of positive correlation between the study variable and the auxiliary variable.

The following table can be compared with **Table 2** of the manuscript where the corresponding correlation coefficient is positive under the same seeds of R commands given in the appendix of the manuscript.

| RE of proposed class of estimators for Situation-I when $k=3$ | | | | | | | | | | |
| --- | --- | --- | --- | --- | --- | --- | --- | --- | --- | --- |
|  | | $\rho=-0.10$ | | | $\rho=-0.50$ | | | $\rho=-0.90$ | | |
| $r'_{2}$ | Est | $\nu=3$ | $\nu=4$ | $\nu=5$ | $\nu=3$ | $\nu=4$ | $\nu=5$ | $\nu=3$ | $\nu=4$ | $\nu=5$ |
| 1 | $t_{r1}$ | 1.209 | 1.283 | 1.271 | 1.311 | 1.372 | 1.355 | 1.316 | 1.365 | 1.364 |
|  | $t_{r2}$ | 4.708 | 6.600 | 7.014 | 5.023 | 7.784 | 9.972 | 5.416 | 8.949 | 10.308 |
|  | $t_{r3}$ | 2.973 | 3.743 | 3.680 | 5.232 | 10.165 | 10.356 | 6.586 | 9.257 | 11.636 |
| 2 | $t_{r1}$ | 1.280 | 1.261 | 1.271 | 1.327 | 1.336 | 1.394 | 1.336 | 1.356 | 1.374 |
|  | $t_{r2}$ | 6.181 | 8.881 | 9.373 | 11.410 | 13.897 | 17.334 | 10.675 | 11.419 | 15.390 |
|  | $t_{r3}$ | 4.554 | 5.434 | 6.172 | 8.336 | 12.410 | 15.366 | 9.355 | 13.122 | 17.160 |
| 3 | $t_{r1}$ | 1.262 | 1.263 | 1.270 | 1.355 | 1.346 | 1.391 | 1.411 | 1.439 | 1.411 |
|  | $t_{r2}$ | 9.439 | 10.660 | 12.231 | 10.933 | 13.901 | 17.434 | 14.691 | 17.150 | 18.111 |
|  | $t_{r3}$ | 5.933 | 7.215 | 8.024 | 8.839 | 15.865 | 19.298 | 11.178 | 17.503 | 18.150 |

2) The simulation results are described very briefly. It definitely needs more elaboration.

**AUTHOR’S RESPONSE:**

Both authors accept to take the reviewers advice, and the simulation results were elaborated carefully. Below is description of simulation results, both before and after the corrections.

| **Before correction** | **After correction** |
| --- | --- |
| simulation results in Table 1-6 shows that the relative efficiency of proposed estimators increases as the value of correlation coefficient increase. the relative efficiency also increases with the increase in overall sample size n, while relative efficiency is observed highest when moderate set size ν = 4 is used. However, the relative efficiency decreases when the value of non-response rate K is increased. | Simulation results in Tables 1-6 show that the RE of the proposed class of estimators under RSS is higher than its competing estimators under SRS for estimating population mean. It is also observed that the RE increases when the correlation coefficient between *Y* and *X* is increased. The RE also increases by increasing the overall sample size *n*, while it decreases when the value of non-response rate *k* is increased. We also observed that the RE is comparatively higher for a moderate set size *v*, i.e., *v=4*. Furthermore, the RE is higher for Situation-I when compared to Situation-II of sampling selection under RSS procedure for the same sample size. |

3) When an acronym (like RSS) is introduced, then it should be used instead of the full form. This simple rule has been violated in the text.

**AUTHOR’S RESPONSE:**

Both authors are thankful to the reviewer for reminding this basic rule. The manuscript is revised and fixed for the issue of all acronyms including RSS.

4) A paragraph should be added to the beginning of Conclusion that outlines what has been done.

**AUTHOR’S RESPONSE:**

The reviewer's insightful comment has been accepted and both authors are thankful for it. The recommendation to add a paragraph outlining what has been done, to the beginning of conclusions is added. Below is the conclusion before and after corrections.

| **Before correction** | **After correction** |
| --- | --- |
| From the simulation results it is observed that the proposed class of estimators is provides more precise results for estimating population mean when non-response occurs in surveys. Furthermore, relative efficiency of proposed class of estimators is higher when RSS is used to collect data at both attempts than using RSS for data collection only on second attempt. So, it is suggested to use the proposed class of estimators under RSS sampling technique. The use of RSS on both attempts of data collection is recommended for the more gain in efficiency of estimates. | In this paper, we proposed a generalized class of Rao-regression type estimators for finite population mean under non-response when RSS is used to collect the data at the second attempt and at both attempts. Expressions for bias and MSE of the proposed class of estimators were derived up to the first order of approximation. A brief simulation study was conducted to determine the RE of the proposed class of estimators for different values of non-response rate *K*, set size *v*, and overall sample size *n*.  Based on simulation results, we suggest using the proposed class of estimators under RSS for more precise estimation of the finite population mean when non-response occurs in the data. Additionally, we advise using the RSS technique for data collection at both attempts. |

5) There is some room to improve English writing. Some specific corrections are highlighted in the attached pdf file (I strongly recommend authors to use language editing service offered by academic centers). Also, there are some problems in the Reference section. Please carefully fix them.

**AUTHOR’S RESPONSE:**

Both authors are very thankful to the reviewer for highlighting issues in the manuscript, specifically related to English writing. The English writing has improved, and all the corrections highlighted by the reviewer are fixed. The English grammar and punctuation are also fixed with the help of a language editing service.

Some changes are given in the following Table

| **Before correction** | **After correction** |
| --- | --- |
| his research area | His research area |
| One of his paper | One of his papers |
| Phd student | PhD student |
| population is to be separated | population to be divided |
| shows population units | be population units |
| Ranked set sampling (RSS) [Heading] | RSS procedure |
| ranked set sampling [acronym] | RSS |
| Eq (1) | Equation (1) |
| can be written as; [drop “;”] | can be written as |
| is given by, [drop “,”] | is given by |
| is given as, [drop “,”] | is given as |
| in the context of assumptions made by | with the same setup used by |
| tables | Tables |
| Proposed class of estimators is provides more precise results for estimating population mean when non-response occurs in surveys.  [drop “is”] | [Whole sentence is changed to the following paragraph]  In this paper, we proposed a generalized class of Rao-regression type estimators for finite population mean under non-response when RSS is used to collect the data at the second attempt and at both attempts. Expressions for bias and MSE of the proposed class of estimators were derived up to the first order of approximation. A brief simulation study was conducted to determine the RE of the proposed class of estimators for different values of non-response rate *K*, set size *v*, and overall sample size *n*. |
| The use of RSS on both attempts of data collection is recommended for the more gain in efficiency of estimates. | Additionally, we encourage using the RSS technique for data collection in both attempts since it provides more precise estimates. |

In addition, the references are revised and corrected carefully. The following references are also added to the manuscript in order to enrich the literature with some applications of RSS techniques.

Murray, R. A., Ridout, M. S., & Cross, J. V. (2000). The use of ranked set sampling in spray deposit assessment. *Aspects of Applied Biology*, *57*, 141-146.

Cobby, J. M., Ridout, M. S., Bassett, P. J., & Large, R. V. (1985). An investigation into the use of ranked set sampling on grass and grass‐clover swards. *Grass and Forage Science*, *40*(3), 257-263.

Chen, Z., Bai, Z., & Sinha, B. K. (2004). *Ranked set sampling: theory and applications* (Vol. 176). New York: Springer.

Samawi, H. M., Al-Saleh, M. F., & Al-Saidy, O. (2008). On the matched pairs sign test using bivariate ranked set sampling: an application to environmental issues. *African Journal of Environmental Science and Technology*, *2*(1), 001-009.

Husby, C. E., Stasny, E. A., & Wolfe, D. A. (2005). An application of ranked set sampling for mean and median estimation using USDA crop production data. *Journal of agricultural, biological, and environmental statistics*, *10*(3), 354-373.

**Reviewer #4:**

Paper ID: PONE-D-22-15506R1

Report of the paper “An efficient class of estimators for finite population mean in the presence of non-response under ranked set sampling (RSS)” Submitted to PLOS ONE
Referee’s Comments

In this paper, authors have suggested a class of Rao-regression type estimators and ranked set sampling (RSS) scheme is used to collect data from different situations non-response. Expressions for bias and mean square error of the estimators are obtained up to first order of approximation and comprehensive simulation study is carried out to observe the performances of the proposed estimators under non-response. Authors have incorporated all the comments raised by the previous reviewer and I agreed with the response. The idea/concept of the paper is interesting and can be published after some corrections. Some suggestions are as follows:

1) I would like to suggest the authors to make an Appendix to post all codes related to this paper in the next submission.

**AUTHOR’S RESPONSE:**

Both authors agree with the reviewer's suggestion, and the R commands used for the simulation study are include in the manuscript. Below is a screenshot of the first few lines.

2) Add these references in the reference section as well in the text section

**a.** Khalid (2019). Effective Estimation Strategy of Population Variance in Two-Phase Successive Sampling Under Random Non-response. J Stat Theory Pract 13, 4 <https://doi.org/10.1007/s42519-018-0010-y>
**b.** Mohd Khalid. "Exponential chain dual to ratio and regression type estimators of population mean in two-phase sampling." Statistica 75.4 (2015): 379-389.
**c.** Khalid, Mohd. "Some imputation methods to deal with the issue of missing data problems due to random non-response in two-occasion successive sampling." Communications in Statistics-Simulation and Computation (2020): 1-21.

**AUTHOR’S RESPONSE:**

The authors feel happy to include these helpful references in the manuscript since these are recent and very relevant research that are pertinent to the subject matter of our paper. These valuable references are cited in both, the text and the references section of the manuscript as shown below.

**Reviewer #5:**

Review report on “An efficient class of estimators for finite population mean in the presence of non-response under ranked set sampling (RSS)”

The manuscript deals with the problem of estimation of population mean in presence of non-response on study character. The properties of the suggested generalized class of Rao-regression type estimators under RSS in presence of non-response have been studied and efficiency has been compared through simulation study.
Although the problem considered in the manuscript has been found to be interesting and mathematically robust but there is some suspicion of incorrectness or typographical error in some of the expressions, and so minor revision is required.

1. On page 5, the expression shown in equations (13), (18) and (19) appears to be erroneous, it should be verified properly.

**AUTHOR’S RESPONSE:**

Both authors are thankful to the reviewer for pointing out a very significant mistake in the manuscript regarding Eq (13), (18) and (19). All these equations are reviewed and corrected by the authors. The relevant change is also made to Eq (12). The corresponding equations before and after corrections are given below:

**Before corrections**:

$\bar{y}'_{2rss}=\frac{1}{n'_{2}}\sum_{i=1}^{n'_{2}} \bar{y}'_{2(i)}$ (12)

$Var\left(\bar{y}'_{2rss}\right)=\frac{\sigma_{2}^{2}}{n'_{2}}-\frac{1}{n'_{2}^{2}}\sum_{i=1}^{n'_{2}} \Delta'_{2y(i)}^{2}$ (13)

$Var\left(\bar{y}_{rss}^{**}\right)=\frac{\sigma_{y}^{2}}{n}+\frac{W_{2}\left(K-1\right)}{n}\sigma_{2y}^{2}-\frac{1}{r\nu^{2}}\left(\sum_{i=1}^{\nu} \Delta_{y(i)}^{2}+\left(K-1\right)\sum_{i=1}^{\nu} \Delta_{2y[i]}^{2}\right)=V''_{yr}$ (18)

$Var\left(\bar{x}_{rss}^{**}\right)=\frac{\sigma_{x}^{2}}{n}+\frac{W_{2}\left(K-1\right)}{n}\sigma_{2x}^{2}-\frac{1}{r\nu^{2}}\left(\sum_{i=1}^{\nu} \Delta_{x(i)}^{2}+\left(K-1\right)\sum_{i=1}^{\nu} \Delta_{2y[i]}^{2}\right)=V''_{xr}$ (19)

**After corrections:**

$\bar{y'}_{2rss}=\frac{1}{n'_{2}}\sum_{j=1}^{r'_{2}} \sum_{i=1}^{\nu} y'_{2\left(i\right)}$ **(12)**

$Var\left(\bar{y'}_{2rss}\right)=\frac{\sigma_{2y}^{2}}{r'_{2}\nu}-\frac{1}{r'_{2}\nu^{2}}\sum_{i=1}^{\nu} \Delta_{2y\left(i\right)}^{2}$ **(13)**

$Var\left(\bar{y}_{rss}^{**}\right)=\frac{\sigma_{y}^{2}}{n}-\frac{1}{r\nu^{2}}\sum_{i=1}^{\nu} \Delta_{y\left[i\right]}^{2}+\frac{W_{2}\left(K-1\right)}{r\nu}\left(\sigma_{2y}^{2}-\frac{1}{\nu}\sum_{i=1}^{\nu} \Delta_{2y\left[i\right]}^{2}\right)=V''_{yr}$ **(18)**

$Var\left(\bar{x}_{rss}^{**}\right)=\frac{\sigma_{x}^{2}}{n}-\frac{1}{r\nu^{2}}\sum_{i=1}^{\nu} \Delta_{x\left(i\right)}^{2}+\frac{W_{2}\left(K-1\right)}{r\nu}\left(\sigma_{2x}^{2}-\frac{1}{\nu}\sum_{i=1}^{\nu} \Delta_{2x\left(i\right)}^{2}\right)=V''_{xr}$ **(19)**

For each of these corrected equations, proof is given below.

**Proof for Eq (13)**

Recall Eq (12)

$\bar{y'}_{2rss}=\frac{1}{n'_{2}}\sum_{j=1}^{r'_{2}} \sum_{i=1}^{\nu} y'_{2\left(i\right)}=\frac{1}{r'_{2}\nu}\sum_{j=1}^{r'_{2}} \sum_{i=1}^{\nu} y'_{2\left(i\right)}$ (12)

Taking variance on both sides

$$Var\left(\bar{y'}_{2rss}\right)=\frac{1}{\left(r'_{2}\nu\right)^{2}}\sum_{j=1}^{r'_{2}} \sum_{i=1}^{\nu} \sigma_{2y\left(i\right)}^{2}$$

where

$$\sigma_{2y\left(i\right)}^{2}=E\left(Y_{2\left(i\right)}-\mu_{2y\left(i\right)}\right)^{2}$$

$$\sigma_{2y\left(i\right)}^{2}=E\left(Y_{2\left(i\right)}-\mu_{2y}+\mu_{2y}-\mu_{2y\left(i\right)}\right)^{2}$$

$$\sigma_{2y\left(i\right)}^{2}=E\left(\left(Y_{2y\left(i\right)}-\mu_{2y}\right)-\left(\mu_{2y\left(i\right)}-\mu_{2y}\right)\right)^{2}$$

$$\sigma_{2y\left(i\right)}^{2}=E\left(Y_{2y\left(i\right)}-\mu_{2y}\right)^{2}+E\left(\mu_{2y\left(i\right)}-\mu_{2y}\right)^{2}-2E\left(Y_{2y\left(i\right)}-\mu_{2}\right)\left(\mu_{2y\left(i\right)}-\mu_{2y}\right)$$

$$\sigma_{2y\left(i\right)}^{2}=E\left(Y_{2y\left(i\right)}-\mu_{2y}\right)^{2}+E\left(\mu_{2y\left(i\right)}-\mu_{2y}\right)^{2}-2\left(\mu_{2y\left(i\right)}-\mu_{2y}\right)\left(\mu_{2y\left(i\right)}-\mu_{2y}\right)$$

$$\sigma_{2y\left(i\right)}^{2}=E\left(Y_{2y\left(i\right)}-\mu_{2y}\right)^{2}+\left(\mu_{2y\left(i\right)}-\mu_{2y}\right)^{2}-2\left(\mu_{2y\left(i\right)}-\mu_{2y}\right)^{2}$$

$$\sigma_{2y\left(i\right)}^{2}=E\left(Y_{2y\left(i\right)}-\mu_{2y}\right)^{2}-\left(\mu_{2y\left(i\right)}-\mu_{2y}\right)^{2}$$

$$\sigma_{2y\left(i\right)}^{2}=\sigma_{2y}^{2}-\left(\mu_{2y\left(i\right)}-\mu_{2y}\right)^{2}$$

Thus

$$Var\left(\bar{y'}_{2rss}\right)=\frac{1}{\left(r'_{2}\nu\right)^{2}}\sum_{j=1}^{r'_{2}} \sum_{i=1}^{\nu} \left(\sigma_{2y}^{2}-\left(\mu_{2y\left(i\right)}-\mu_{2y}\right)^{2}\right)$$

$$Var\left(\bar{y'}_{2rss}\right)=\frac{\left(r'_{2}\nu\right)\sigma_{2y}^{2}}{\left(r'_{2}\nu\right)^{2}}-\frac{r'_{2}}{\left(r'_{2}\nu\right)^{2}}\sum_{i=1}^{\nu} \left(\mu_{2y\left(i\right)}-\mu_{2y}\right)^{2}$$

$Var\left(\bar{y'}_{2rss}\right)=\frac{\sigma_{2y}^{2}}{r'_{2}\nu}-\frac{1}{r'_{2}\nu^{2}}\sum_{i=1}^{\nu} \Delta_{2y\left(i\right)}^{2}$ **(13)**

where

$$\Delta_{2y\left(i\right)}=\mu_{2y\left(i\right)}-\mu_{y2}$$

$\mu_{2y\left(i\right)}$= Mean of ith order statistics in non-response group for variable *Y*

$\mu_{y2}$ = Population mean of non-responding units for variable *Y*

$\sigma_{2y}^{2}$= population variance of non-responding units for variable *Y*

Eq (13) also can be written as,

$$Var\left(\bar{y'}_{2rss}\right)=\frac{\sigma_{2y}^{2}}{n'_{2}}-\frac{1}{n'_{2}\nu}\sum_{i=1}^{\nu} \Delta_{2y\left(i\right)}^{2}$$

**Proof for Eq (18) and Eq (19)**

Recall Eq (17)

$\bar{y}_{rss}^{**}=w_{1}\bar{y}_{1rss}+w_{2}\bar{y}'_{2rss}$ (17)

Eq (17) can be written as,

$$\bar{y}_{rss}^{**}=\left[\left(w_{1}\bar{y}_{1rss}+w_{2}\bar{y}_{2rss}\right)/s\right]+\left[w_{2}\left(\bar{y}'_{2rss}-\bar{y}_{2rss}\right)/s_{2}\right]$$

In above equation, the term $\left(w_{1}\bar{y}_{1rss}+w_{2}\bar{y}_{2rss}\right)/s$ represents a sample of size $n$ selected by method of RSS from the overall population, which has the variance $\frac{\sigma_{y}^{2}}{n}-\frac{1}{n\nu}\sum_{i=1}^{\nu} \Delta_{\left(i\right)}^{2}$ , thus conditional variance of $\bar{y}_{rss}^{**}$can be written as,

$Var\left(\bar{y}_{rss}^{**}\right)=\frac{\sigma_{y}^{2}}{n}-\frac{1}{n\nu}\sum_{i=1}^{\nu} \Delta_{y\left[i\right]}^{2}+w_{2}^{2}E\left(\left(\bar{y}'_{2rss}-\bar{y}_{2rss}\right)/s_{2}\right)^{2}$ (17B)

Variance for the explicit function of second term in the R.H.S is,

$$w_{2}^{2}Var\left[\left(\bar{y}'_{2rss}-\bar{y}_{2rss}\right)/s_{2}\right]=w_{2}^{2}E\left(\left(\bar{y}'_{2rss}-\bar{y}_{2rss}\right)/s_{2}\right)^{2}$$

$$w_{2}^{2}Var\left[\left(\bar{y}'_{2rss}-\bar{y}_{2rss}\right)/s_{2}\right]=w_{2}^{2}E\left(\left(\bar{y}'_{2rss}-\mu_{2}\right)-\left(\bar{y}_{2rss}-\mu_{2}\right)/s_{2}\right)^{2}$$

$$w_{2}^{2}Var\left[\left(\bar{y}'_{2drss}-\bar{y}_{2drss}\right)/s_{2}\right]=w_{2}^{2}E\left(\left(\bar{y}'_{2rss}-\mu_{2}\right)^{2}/s_{2}+\left(\bar{y}_{2rss}-\mu_{2}\right)^{2}/s_{2}-2\left(\bar{y}'_{2rss}-\mu_{2}\right)\left(\bar{y}_{2rss}-\mu_{2}\right)/s_{2}\right)^{2}$$

Conditioning third term to sample of size $n_{2}$

$$w_{2}^{2}Var\left[\left(\bar{y}'_{2rss}-\bar{y}_{2rss}\right)/s_{2}\right]=w_{2}^{2}E\left(\left(\bar{y}'_{2rss}-\mu_{2}\right)^{2}/s_{2}-\left(\bar{y}_{2rss}-\mu_{2}\right)^{2}/s_{2}\right)$$

$$w_{2}^{2}Var\left[\left(\bar{y}'_{2rss}-\bar{y}_{2rss}\right)/s_{2}\right]=w_{2}^{2}\left(Var\left(\bar{y}'_{2rss}\right)/s_{2}-Var\left(\bar{y}_{2rss}\right)/s_{2}\right)$$

$$w_{2}^{2}Var\left[\left(\bar{y}'_{2rss}-\bar{y}_{2rss}\right)/s_{2}\right]=w_{2}^{2}\left[\frac{\sigma_{2y}^{2}}{n'_{2}}-\frac{1}{n'_{2}\nu}\sum_{i=1}^{\nu} \Delta_{2y\left[i\right]}^{2}-\frac{\sigma_{2y}^{2}}{n_{2}}+\frac{1}{n_{2}\nu}\sum_{i=1}^{\nu} \Delta_{2y\left[i\right]}^{2}\right]$$

$$w_{2}^{2}Var\left[w_{2}\left(\bar{y}'_{2rss}-\bar{y}_{2rss}\right)/s_{2}\right]=w_{2}^{2}\left(\frac{\sigma_{2y}^{2}}{n'_{2}}-\frac{\sigma_{2y}^{2}}{n_{2}}\right)-w_{2}^{2}\left[\frac{1}{n'_{2}\nu}\sum_{i=1}^{\nu} \Delta_{2y\left[i\right]}^{2}-\frac{1}{n_{2}\nu}\sum_{i=1}^{\nu} \Delta_{2y\left[i\right]}^{2}\right]$$

$$w_{2}^{2}Var\left[w_{2}\left(\bar{y}'_{2rss}-\bar{y}_{2rss}\right)/s_{2}\right]=w_{2}^{2}\left(\frac{k\sigma_{2y}^{2}}{n_{2}}-\frac{\sigma_{2y}^{2}}{n_{2}}\right)-w_{2}^{2}\left[\frac{k}{n_{2}\nu}\sum_{i=1}^{\nu} \Delta_{2y\left[i\right]}^{2}-\frac{1}{n_{2}\nu}\sum_{i=1}^{\nu} \Delta_{2y\left[i\right]}^{2}\right]$$

$$w_{2}^{2}Var\left[w_{2}\left(\bar{y}'_{2rss}-\bar{y}_{2rss}\right)/s_{2}\right]=\frac{w_{2}^{2}\left(k-1\right)}{n_{2}}\sigma_{2y}^{2}-\frac{w_{2}^{2}}{n_{2}\nu}\left[k\sum_{i=1}^{\nu} \Delta_{2y\left[i\right]}^{2}-\sum_{i=1}^{\nu} \Delta_{2y\left[i\right]}^{2}\right]$$

$$w_{2}^{2}Var\left[w_{2}\left(\bar{y}'_{2rss}-\bar{y}_{2rss}\right)/s_{2}\right]=\frac{w_{2}^{2}\left(k-1\right)}{n_{2}}\sigma_{2y}^{2}-\frac{w_{2}^{2}\left(k-1\right)}{n_{2}\nu}\left[\sum_{i=1}^{\nu} \Delta_{2y\left[i\right]}^{2}\right]$$

$$w_{2}^{2}Var\left[w_{2}\left(\bar{y}'_{2rss}-\bar{y}_{2rss}\right)/s_{2}\right]=\frac{w_{2}\left(k-1\right)}{n}\sigma_{2y}^{2}-\frac{w_{2}\left(k-1\right)}{n\nu}\left[\sum_{i=1}^{\nu} \Delta_{2y\left[i\right]}^{2}\right]$$

$$w_{2}^{2}Var\left[w_{2}\left(\bar{y}'_{2rss}-\bar{y}_{2rss}\right)/s_{2}\right]=\frac{w_{2}\left(k-1\right)}{n}\left(\sigma_{2y}^{2}-\frac{1}{\nu}\sum_{i=1}^{\nu} \Delta_{2y\left[i\right]}^{2}\right)$$

Eq (17B) becomes

$$Var\left(\bar{y}_{rss}^{**}\right)=\frac{\sigma_{y}^{2}}{n}-\frac{1}{n\nu}\sum_{i=1}^{\nu} \Delta_{y\left[i\right]}^{2}+\frac{w_{2}\left(k-1\right)}{n}\left(\sigma_{2y}^{2}-\frac{1}{\nu}\sum_{i=1}^{\nu} \Delta_{2y\left[i\right]}^{2}\right)$$

Thus, expected variance of $\bar{y}_{rss}^{**}$ is given by

$Var\left(\bar{y}_{rss}^{**}\right)=\frac{\sigma_{y}^{2}}{n}-\frac{1}{r\nu^{2}}\sum_{i=1}^{\nu} \Delta_{y\left[i\right]}^{2}+\frac{W_{2}\left(K-1\right)}{r\nu}\left(\sigma_{2y}^{2}-\frac{1}{\nu}\sum_{i=1}^{\nu} \Delta_{2y\left[i\right]}^{2}\right)=V''_{yr}$ **(18)**

similar result can be obtained for the auxiliary variable *X* as,

$Var\left(\bar{x}_{rss}^{**}\right)=\frac{\sigma_{x}^{2}}{n}-\frac{1}{r\nu^{2}}\sum_{i=1}^{\nu} \Delta_{x\left(i\right)}^{2}+\frac{W_{2}\left(K-1\right)}{r\nu}\left(\sigma_{2x}^{2}-\frac{1}{\nu}\sum_{i=1}^{\nu} \Delta_{2x\left(i\right)}^{2}\right)=V''_{xr}$ **(19)**

2. On page 6, "RSS only in second attempt and both attempts" the situation should be corrected.

**AUTHOR’S RESPONSE:**

Thank you for pointing this issue. The situations are corrected correspondingly from

to
